# Antitumor effect and metabonomics of niclosamide micelles

**DOI:** 10.1111/jcmm.17509

**Published:** 2022-08-03

**Authors:** Jiarong Hang, Yu Chen, Lukuan Liu, Liwen Chen, Jiqin Fang, Fei Wang, Miao Wang

**Affiliations:** ^1^ School of Life Science and Biopharmaceutics Shenyang Pharmaceutical University Shenyang China; ^2^ Faculty of Robot Science and Engineering Northeastern University Shenyang China

**Keywords:** cytotoxicity, metabonomics, nanomicelle, niclosamide

## Abstract

Polymer micelles now have promising applications in the treatment of cancer, increasing the water solubility and bioavailability of drugs. Previous studies have found that micelles of niclosamide have good anti‐liver cancer effect. In view of the poor water solubility of niclosamide (NIC), we decided to prepare niclosamide micelles. However, its therapeutic mechanism is not clear, so this paper conducted a preliminary study on its vitro anti‐tumour mechanism and metabonomics to find out its impact. It was found that the drug‐loaded micelles (PEG_2K_‐FIbu/NIC) had an inhibitory effect on HepG2 cells. Moreover, it can promote apoptosis of HepG2 cells and block S and G2/M phase of cell cycle. The plasma and liver metabolomics of mice in normal group, model group and administration group were studied by UPLC‐MS and ^1^H‐NMR. Principal component analysis (PCA) and orthogonal partial least squares discriminant analysis (OPLS‐DA) were used to process the data and find the relevant metabolites. metaboanalyst 5.0 was used to integrate the relevant metabolites to find the main related metabolic pathways. Thus, the anti‐tumour mechanism of PEG_2K_‐FIbu/NIC was analysed. Fifty‐one biomarkers were detected in plasma, and 43 biomarkers were detected in liver. After comprehensive biomarker and metabolic pathway analysis, it was found that PEG_2K_‐FIbu/NIC micelles could affect the changes of many metabolites, mainly affecting amino acid metabolism. This article is an in‐depth study based on the published *Preparation and pharmacodynamics of niclosamide micelles* (DOI: 10.1016/j.jddst.2021.103088).

## 1 INTRODUCTION

Liver cancer is one of the most common and deadly cancers today. Chemotherapy is a common treatment for liver cancer.^1^ Nanocarriers have become an area of research in cancer therapy. The poor water solubility of niclosamide limits its clinical application. However, polymer micelles are a new drug delivery system for cancer treatment and achieved great success in clinical research; it is one of the most promising nanomicelles. The usefulness of polymer micelles is often demonstrated in tumour mouse models. They have good biocompatibility and degradability in vivo, prolong circulation time and reduce toxic side effects. Therefore, micelles of niclosamide were prepared to increase its water solubility and antitumor effect.

Systems biology includes genomics, transcriptomics, proteomics and metabolomics. Metabolomics is the parsing of metabolites in cells and tissues, and is used as a tool for biomarker discovery to understand the mechanism of disease by detecting changes in biological pathways.^2^ Metabolomics is highly sensitive and highly expressed, which can be used to characterize a large number of metabolites in cells and tissues in high throughput. Understanding metabolism can provide therapeutic targets and promote research and development of cancer therapies and drugs.^3^ The number of human metabolites is unclear. Metabolomics includes metabolic fingerprinting, metabolic analysis and isotope‐based target analysis.^4^ Metabolomics has officially become an increasingly popular tool in the life sciences, NMR has been used for metabolic research and analysis, and this connection is increasingly close; NMR has unique advantages in describing the chemical components in mixtures.^5^ UPLC‐MS greatly improves coverage, reduces analysis time, improves separation efficiency, and improves both separation degree and sensitivity with many examples showing that it has been applied in different cancers to obtain potential biomarkers.^6^ Liver cancer is the most common cancer worldwide and the third leading cause of cancer‐related death. Liver cancer is comprised of mainly hepatocellular carcinoma and a few rare liver cancers. In the advanced stage of liver cancer, there is no effective method, so biomarkers are needed as therapeutic targets.^7^ Molecular biomarkers have high sensitivity and specificity and can be used for early diagnosis of cancer.^8^

We prepared PEG_2K_‐Fmoc‐Ibuprofen micelle (PEG_2K_‐FIbu); it consists of PEG hydrophilic segments, Fmoc motifs and ibuprofen hydrophobic domains. Polyethylene glycol (PEG) as a hydrophilic shell, Fmoc can improve drug loading capacity,^9^, ^10^ ibuprofen can increase the sensitivity of tumours to anticancer drugs and thus increase the antitumor activity of niclosamide. In this paper, we first studied the in vitro anti‐tumour mechanism of niclosamide micelles, and then carried out metabolic studies, which were analysed by ^1^H‐NMR and UPLC‐MS techniques to find relevant biomarkers and metabolic pathways, so as to provide the basis for prevention.

## 2 MATERIALS AND METHODS

### 2.1 Mice

Healthy Balb/c male mice and Kunming male mice with a body weight of 18 ± 2 g were provided by the Animal Center of Shenyang Pharmaceutical University. The mice were exposed to light (12 h light/12 h dark), temperature (23 ± 3°C), humidity (40%–70%), and normal diet and free drinking water 1 week before the experiment. All animal experiments were approved by the Medical Ethics Committee of Shenyang Pharmaceutical University.

### 2.2 Cells

H22 cells were purchased from Guangzhou Cellcook Biotech Co., Ltd. Human. Human HepG2 cells were purchased from Guangzhou Cellcook Biotech Co., Ltd. Human. RPMI 1640 medium containing 10% foetal bovine serum, penicillin and streptomycin were been used to culture the HepG2 cells in an incubator at 37°C and 5% CO_2_.

### 2.3 Chemicals and reagents

Heavy water and TSP were purchased from Merck in Germany. Chromatographic pure acetonitrile was purchased from Fisher Company, USA. Chromatographic pure formic acid was purchased from Tianjin Kemiou Chemical Reagent Co., LTD. PEG_2K_‐FIbu and PEG_2K_‐FIbu/NIC were self‐made in the laboratory.

### 2.4 Study on antitumor mechanism in vitro

#### 2.4.1 Cell culture

Cells were propagated in RPMI 1640 media containing 10% foetal bovine serum and 1% antibiotics in 5% CO_2_ at 37°C.Standard cell line was cultivated and cryopreserved according to the producer guideline; cells were used when 80% confluence.

#### 2.4.2 MTT assay

Standard cell line was cultivated and cryopreserved according to the producer guideline; cells were used when 80% confluence. Briefly, the exponentially growing cells (1 × 10^4^ cells/well) were seeded in 96‐well plates. After 24 h incubation, the cells were treated with various concentrations of drug. After treatment for 24 and 48 h, respectively, the 10 μl MTT was added to each well for 4 h incubation at 37°C. Then took out the 96‐well plate and carefully discarded the supernatant of each well to avoid absorbing the crystalline material out. 150 μl DMSO was added to each well, and the crystal was dissolved by low‐speed oscillation for 10 min. The absorbance value was determined at the wavelength of 490 nm with a microplate reader. The experimental groups were as follows: (1): medication group (20, 50, 100, 300, 1000, 3000 ng/mL); (2): blank group; (3): negative control group.

#### 2.4.3 Apoptosis assay

Cells at logarithmic growth stage were inoculated into 6‐well plates with a cell density of 5 × 10^5^. After incubation in an incubator for 24 h, they were administered for 48 h. After reaching the time point, the culture medium was recycled into the 15 ml centrifuge tube, and the 6‐well plate was cleaned with appropriate PBS. Trypsin without EDTA was added to each well and digested in the incubator for 2 min. Culture medium was added to each well to terminate digestion. Then, the cells were placed in an incubator for 30 min, and the cell suspension was mixed with the recovered culture medium and centrifuged at 1000 *r*/min for 5 min, and the supernatant was discarded. The cell precipitates were suspended with 1 ml pre‐cooled PBS, centrifuged at 1000 *r*/min for 5 min and washed twice. The cells were suspended with 100 μl 1 × Binding buffer, 5 μl Annexin V‐FITC and 5 μl PI added into the centrifuge tube. The cells were mixed gently and incubated at room temperature for 15 min away from light. After staining and incubation, 400 μl of 1 × Binding buffer was added to each tube, and the mixture was detected by flow cytometry. Repeat the experiment three times.

#### 2.4.4 Cell cycle assay

Cells at logarithmic growth stage were inoculated into 6‐well plates with a cell density of 5 × 10^5^. After incubation in an incubator for 24 h, they were administered for 48 h. After reaching the time point, the culture medium was recycled into a 15 ml centrifuge tube, the 6‐well plate was cleaned with appropriate PBS, and trypsin was added to each well for digestion in the incubator for 3 min. Digestion was terminated with the recovered culture medium. Centrifugation, 1000 *r*/min, 5 min, supernatant was discarded. 1 ml pre‐cooled PBS was added for re‐suspension, centrifugation, 1000 *r*/min, 5 min, supernatant was discarded, and the bottom of the centrifuge tube was gently slammed to disperse cells appropriately to avoid cell clumping. 1 ml pre‐cooled PBS was added for re‐suspension, vortex was added, and 3 ml pre‐cooled 75% ethanol was added for re‐suspension at 4°C overnight. Centrifugation, 1000 *r*/min, 5 min, supernatant was discarded. Add 1 ml pre‐cooled PBS to resuscitate the cells and discard the supernatant. Add 500 μl PI to suspension, and bath at 37°C for 30 min. Flow cytometry was used.

### 2.5 Sample pretreatment

#### 2.5.1 ^1^H‐NMR Sample pretreatment

H22 liver samples: Samples should be thawed at room temperature before analysis. 1 g liver sample was taken, 1.7 ml normal saline was added to homogenate and centrifuged at 13,000 *r*/min for 10 min, and supernatant was taken as homogenate.

Took 400 μl homogenate, added 400 μl NaH_2_PO_4_‐Na_2_HPO_4_ (0.2 M, pH 7.4) buffer, scrolled for 30 s, centrifugation, 13,000 *r*/min, 10 min, took 500 μl supernatant, added 1 mg/ml TSP heavy water 150 μl, scrolled for 30 s.

Plasma samples: Samples were thawed at room temperature before analysis. Took 400 μl plasma, added 200 μl NaH_2_PO_4_‐Na_2_HPO_4_ (0.2 M, pH 7.4) buffer, scrolled for 30 s, centrifuged, 13,000 *r*/min, 10 min, took supernatant 550 μl, added 1 mg/ ml TSP heavy water 100 μl, scrolled for 30 s.

#### 2.5.2 UPLC‐MS Sample pretreatment

H22 liver samples: Took 100 μl homogenate, added 600 μl acetonitrile to precipitate protein, scrolled for 30 s, centrifuged, 13,000 *r*/min, 10 min, and filtrated with 0.22 μm microporous membrane.

Plasma samples: 200 μl plasma was collected, 600 μl acetonitrile was added to precipitate protein, and the plasma was filtered through 0.22 μm microporous membrane for 30 s.

### 2.6 ^1^H‐NMR data collection

The analysis was performed on Bruker AV 600 MHz superconducting Fourier transform nuclear magnetic resonance spectrometer. The plasma and liver samples were tested by relaxation‐edited pulse train (CPMG). TSP was taken as the reference peak of chemical shift, which was set as δ 0 ppm. One dimensional NMR spectra were obtained from the free induction attenuated signals by Fourier transform.

### 2.7 UPLC‐MS analysis conditions

#### 2.7.1 The chromatographic conditions

Chromatographic column: Universil XB C18 column (150 mm × 2.1 mm, 1.8 μm; Kromat). Mobile phase: 0.1% formic acid–water (1); 0.1% formic acid ‐ acetonitrile (2). Flow rate: 0.2 ml/min. Column temperature: 35°C. Sample room temperature: 4°C. Injection volume: 5 μl. Elution gradient is shown in Tables 1 and 2 (A._1_, A._2_).

**TABLE 1:** (A._1_) Plasma elution gradient

Time (min)	A (%)
0	95
8	69
13	35
22	15
24	15
25	95
30	95

**TABLE 2:** (A._2_) Liver elution gradient

Time (min)	A (%)
0	95
8	69
15	20
16	95
20	95

#### 2.7.2 Mass spectrometry conditions

The ion source was electrospray ionization source (ESI), the positive ion detection mode, the capillary voltage was 3.2 kV, the cone hole voltage was 35 V, the ion source temperature was 120°C, the desolvent temperature was 350°C, the desolvent gas flow rate was 500 L/h, the cone hole gas flow rate was 30 L/h, the collision gas was argon, and the full scan and sub‐ion scan were used. The full scan quality range m/z is 100–1000 Da.

#### 2.7.3 UPLC‐MS methodological analysis

Equal volumes of plasma to be tested were mixed separately to make mixed plasma samples for quality control (QC), and liver QC samples were prepared in the same way. Six different peaks on the chromatogram were selected, and the reproducibility, system stability, sample pretreatment stability and sample freeze–thaw cycle stability of the method were investigated by calculating the relative standard deviation (RSD) or relative error (RE) of the retention time (t_R_) and the corresponding peak area.

### 2.8 Data processing

#### 2.8.1 ^1^H‐NMR data processing

The ^1^H‐NMR spectra of the samples were imported into The mestrenova 6.1 software. Phase adjustment and baseline correction were performed with the TSP chemical shift (δ 0 ppm) as a reference, and the spectra between 0 and 10 ppm were piecewise integrated according to the width of 0.04 ppm. After normalized processing, the data were imported into simca‐p 13.0 (Umetrics, Umea) for multivariate statistical analysis by orthogonal partial least squares discrimination analysis (OPLS‐DA) method. Metabolites meeting both *VIP* > 1.0 and *p* < 0.05 were the final biomarkers.

#### 2.8.2 UPLC‐MS data processing

In markerlynx V4.1 software, chromatogram peak recognition, peak matching and normalization were performed on the original atlas and then imported into simca‐p 13.0 (Umetrics, Umea) software. Orthogonal partial least squares discriminant analysis (OPLS‐DA) was used to analyse the metabolite spectra of plasma and liver tissue, and the score and loading plots were obtained. The threshold value of variable importance is greater than 1.0, indicating that the metabolites are different in multidimensional statistics, and t‐test statistical analysis is carried out. When *p* < 0.05, it is considered that the metabolites are different in one‐dimensional, and the metabolites that meet both *VIP* > 1.0 and *p* < 0.05 are the final differential metabolites. The biomarkers were identified by positive and negative ion first order and selective ion second order mass spectrometry, combined with the literature and HMDB online database (http://www.hmdb.ca/).

The main parameters of markerlynx software: Initial retention time and final retention time were 0 and 30 min, respectively; initial retention time and final retention time of the liver were 0 and 20 min, respectively; the low mass and high mass were 100 and 1000 Da, respectively. Mass number error (mass tolerance) of mass spectrum was 0.05 Da. The noise elimination level is 6.00.

## 3 RESULTS AND DISCUSSION

### 3.1 MTT assay

**FIGURE 1:**
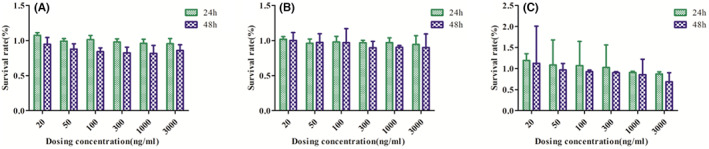
Effect of drugs on HepG2 cells showed that PEG_2K_‐FIbu had almost no toxicity to cells, while PEG_2K_‐FIbu/NIC had no toxicity to HepG2 cells. (A) NIC (B) PEG_2K_‐FIbu (C) PEG_2K_‐FIbu/NIC

MTT assay was used to determine the toxicity of NIC, PEG_2K_‐FIbu and PEG_2K_‐FIbu/NIC to HepG2 cells treated for 24 and 48 h, respectively. The cytotoxicity of NIC and PEG_2K_‐FIbu/NIC increased with the increase of dose and the prolongation of action time, indicating that both NIC and PEG_2K_‐FIbu/NIC have dose‐dependent and time‐dependent inhibition effects on HepG2 cells. Blank carrier should be non‐toxic, low toxicity to ensure the safety of the human body, the cell survival rate increased with the increase of blank carrier concentration decreased, and the maximum concentration of 3000 ng/ml, the cell survival rate is still above 80%, blank carrier is safe and has good biocompatibility with human body, for drug micelle less toxicity, can be used as a drug delivery carrier. The toxicity of PEG_2K_‐FIbu/NIC increased with the increase of NIC concentration, indicating that they could release NIC in cells and kill cells. However, because micelles are internally hydrophobic and externally hydrophilic, NIC is wrapped in the interior of micelles, resulting in a slow and lasting release, which increases the residence time of NIC in the body and can better inhibit cell growth. However, free NIC rapidly enters cells through passive diffusion to play its role, so PEG_2K_‐FIbu/NIC can inhibit cell growth better than NIC. Cytotoxicity test results are shown in Figure 1.

### 3.2 Apoptosis assay

**FIGURE 2:**
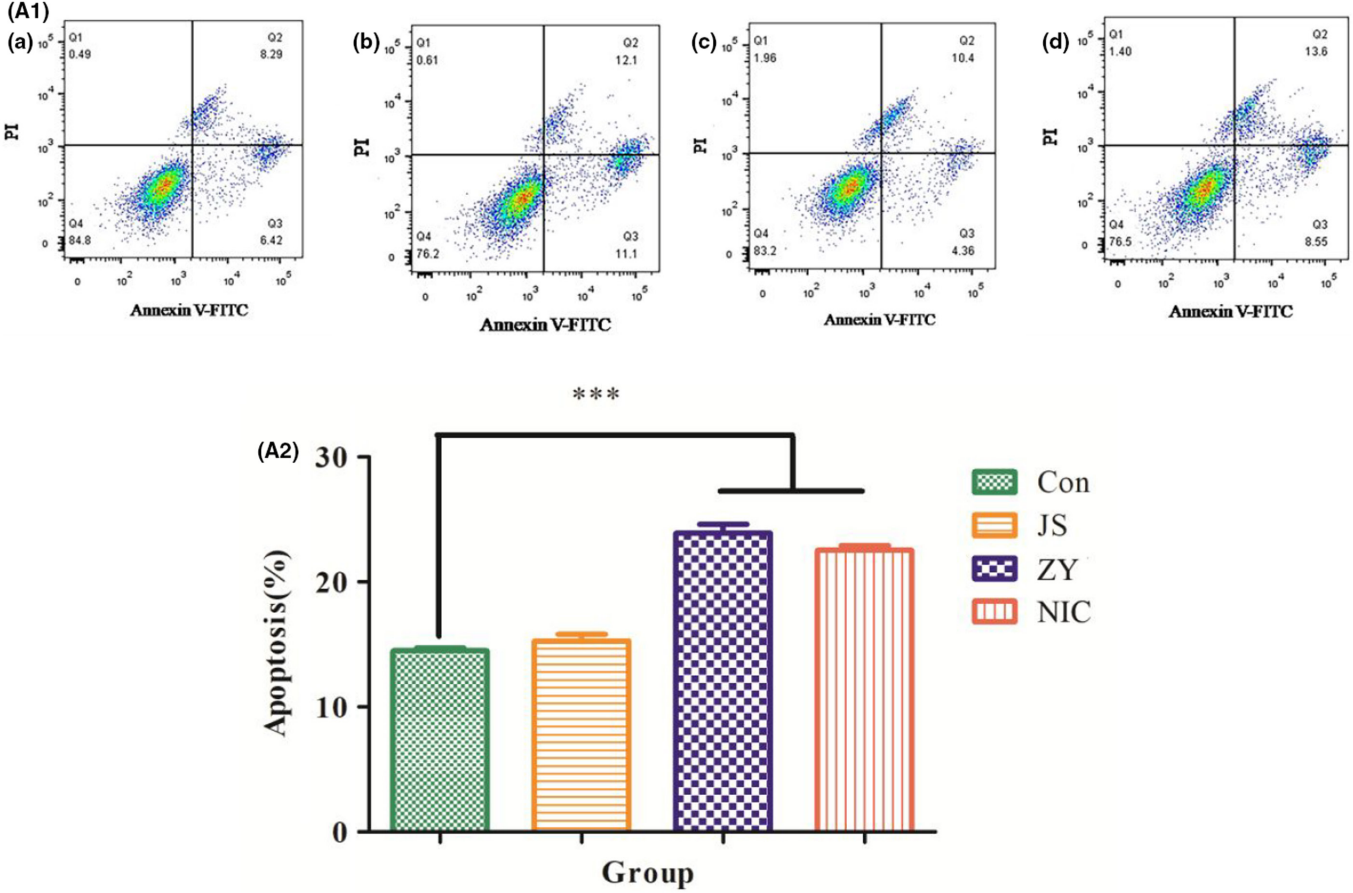
(A_1_) (a) blank group (b) PEG_2K_‐FIbu/NIC (c) PEG_2K_‐FIbu (d) NIC. NIC group of apoptosis rate was 22.15%; PEG_2K_‐FIbu/NIC group of apoptosis rate was 23.2%. (A_2_) PEG_2K_‐FIbu has almost no effect; NIC and PEG_2K_‐FIbu/NIC can induce apoptosis of HepG2 cells

Apoptosis of each group was shown in Figure 2 (A._1_, A._2_). Compared with the blank group, PEG_2K_‐FIbu has almost no effect, NIC and PEG_2K_‐FIbu/NIC can induce apoptosis of HepG2 cells, NIC group of apoptosis rate was 22.15%, PEG_2K_‐FIbu/NIC group of apoptosis rate was 23.2%, and determined by MTT experiment, and compared with significant difference, that can induce cell apoptosis.

### 3.3 Cell cycle assay

**FIGURE 3:**
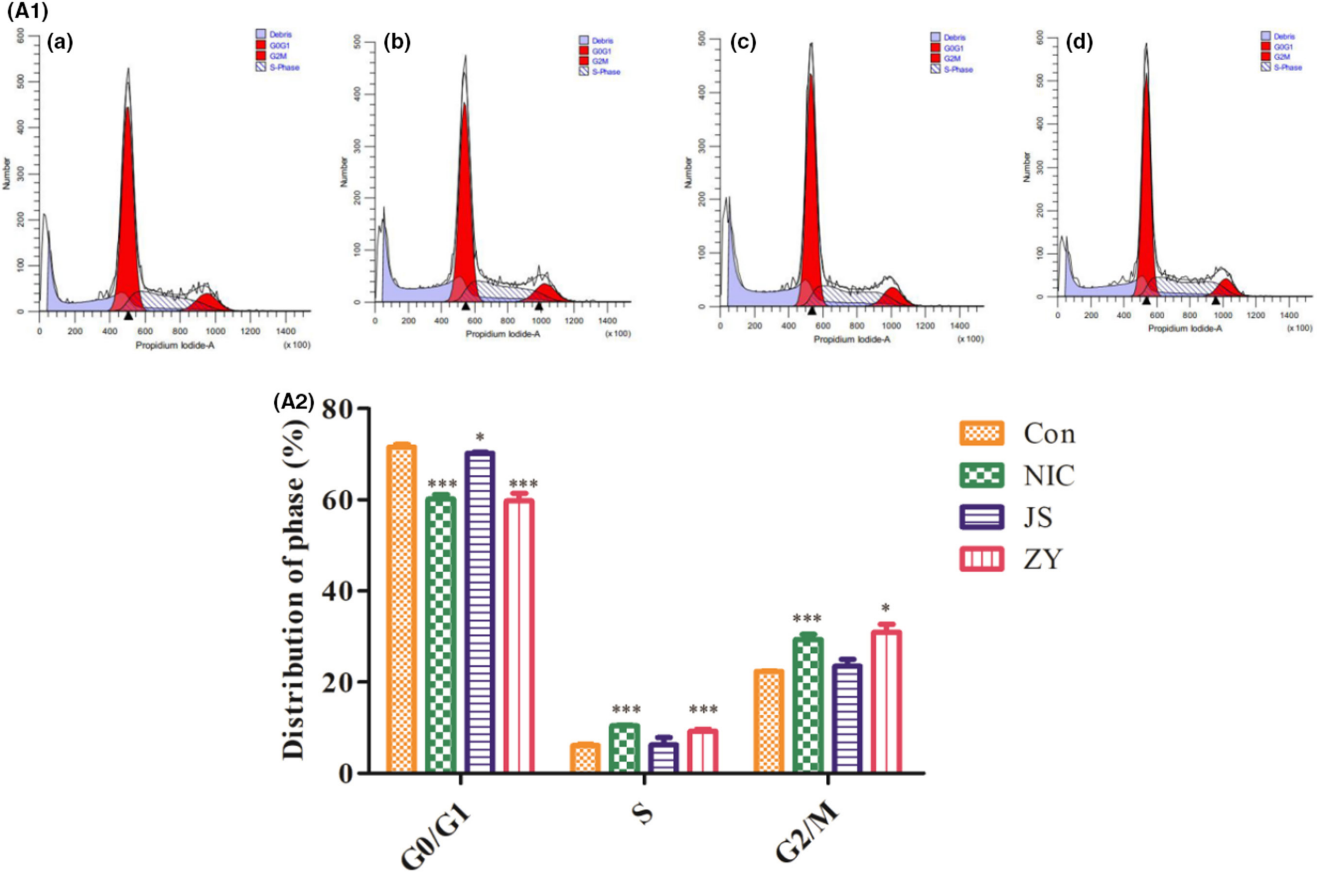
(A_1_) (a) blank group (b) NIC (c) PEG_2K_‐FIbu (d) PEG_2K_‐FIbu/NIC. (A_2_) Drug mainly blocks the S and G2/M phases

Cell cycle diagram is shown in Figure 3 (A._1_, A._2_). It can be seen from the figure that the drug mainly blocks the S and G2/M phases.

### 3.4 ^1^H‐NMR results

The metabolites in plasma and liver detected by NMR are shown in Tables S1 and S2, respectively. NMR profiles of plasma and liver samples are shown in Figure S4 (A._1_, A._2_).

#### 3.4.1 Data analysis

##### Global PCA analysis

**FIGURE 4:**
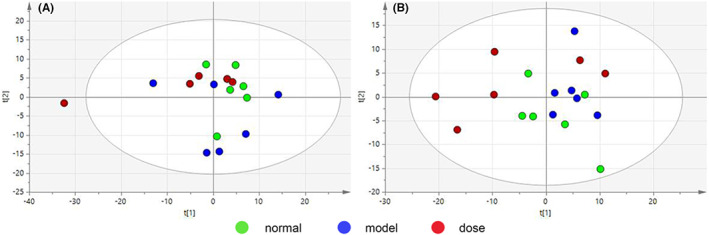
Overall PCA map obtained by NMR technique, (A) plasma group *R* ^*2*^ *X* = 0.945, *Q* ^*2*^ = 0.642; (B) liver group *R* ^*2*^ *X* = 0.768, *Q* ^*2*^ = 0.47

PCA images of plasma and liver are shown in Figure 4, respectively. Plasma sample *R* ^*2*^ *X* = 0.945, *Q* ^*2*^ = 0.642. Liver sample *R* ^*2*^ *X* = 0.768, *Q* ^*2*^ = 0.47. Significant systemic metabolic differences were observed between these groups.

##### OPLS‐DA score plot

**FIGURE 5:**
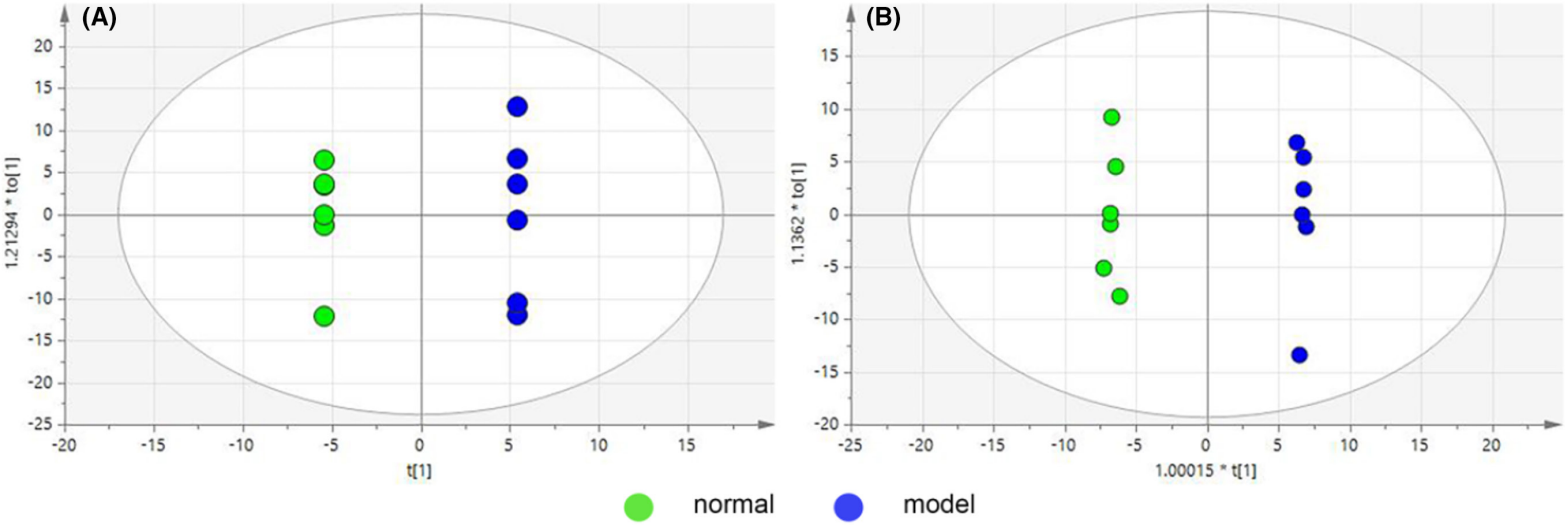
OPLS‐DA score map, obtained by NMR, compares the normal group with the model group. (A) plasma group *R* ^*2*^ *X* = 1, *R* ^*2*^ *Y* = 1, *Q* ^*2*^ = 0.987; (B) liver group *R* ^*2*^ *X* = 0.659, *R* ^*2*^ *Y* = 0.992, *Q* ^*2*^ = 0.883

The score plot is used to show the natural grouping of samples. Each point in the graph represents a sample, and the location of the sample in space is determined by the difference of metabolites contained in the sample. Samples with similar states are more likely to contain the same metabolites. In the score plot, the closer the samples are, while the farther the samples are, the greater the differences in metabolites are. According to this, different groups of samples are classified. OPLS‐DA of simca‐p 13.0 (Umetrics, Umea) software was used to perform pattern recognition (PR) on mouse plasma and liver samples from the normal group, model group and administration group, and the output plasma and liver samples were shown in Figure 5.

The OPLS‐DA model is validated by *R* ^*2*^ *Y* and *Q* ^*2*^ values, where the parameter *R* ^*2*^ *Y* represents whether the model matches the data, and the parameter *Q* ^*2*^ is used to provide an assessment of the model's predictive power. Both *R* ^*2*^ *Y* and *Q* ^*2*^ are greater than 0.5, indicating that the model has good explanatory and predictive rates. The closer the model is to 1, the better its explanatory and predictive capabilities will be. Plasma samples of normal and model group *R* ^*2*^ *X* = 1, *R* ^*2*^ *Y* = 1, *Q* ^*2*^ = 0.987. Liver samples in normal and model group *R* ^*2*^ *X* = 0.659, *R* ^*2*^ *Y* = 0.992, *Q* ^*2*^ = 0.883.

##### OPLS‐DA loading plot

**FIGURE 6:**
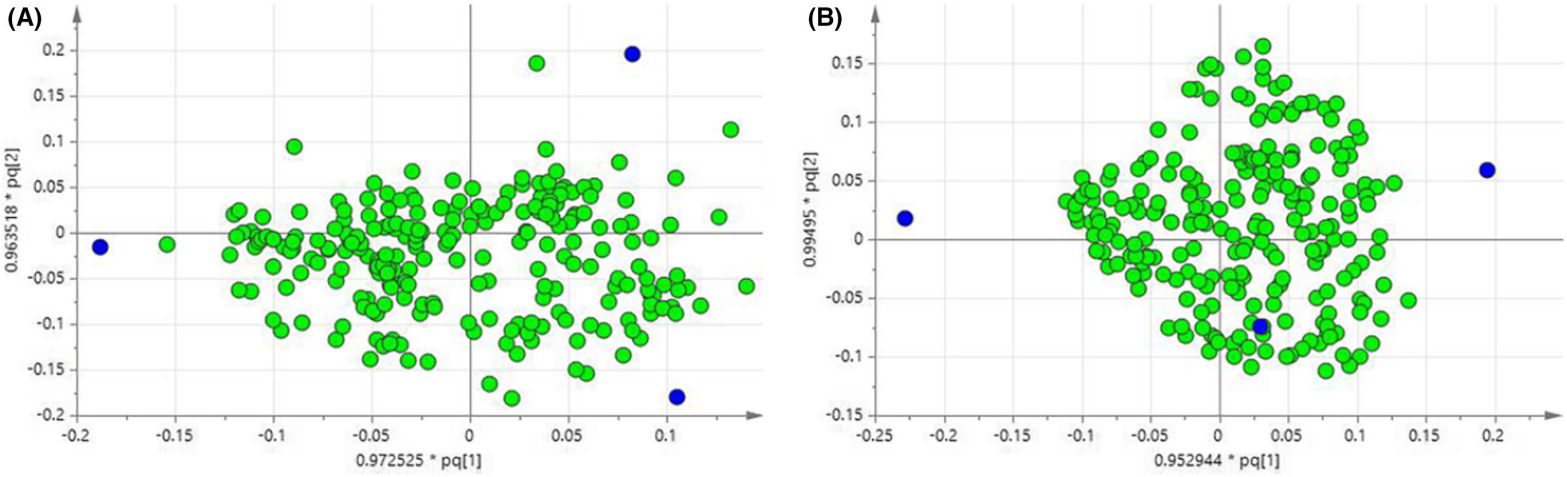
In the Loading diagram obtained by NMR, each point represents a metabolite in the sample. Metabolites farther from the origin are considered to contribute more to sample classification. (A) plasma group; (B) liver group

Loading diagrams are used to find differential variables, with each point representing a metabolite in the sample. Metabolites farther away from the origin are considered to contribute more to sample classification. In the OPLS model, *VIP* > 1.0 was used as the threshold, and spss 19.0 (IBM) software was used for *t*‐test (*p* < 0.05). Metabolites with *VIP* > 1.0 and *p* < 0.05 were selected as the final biomarkers. Loading plots of mouse plasma and liver are shown in Figure 6.

##### Heat map

**FIGURE 7:**
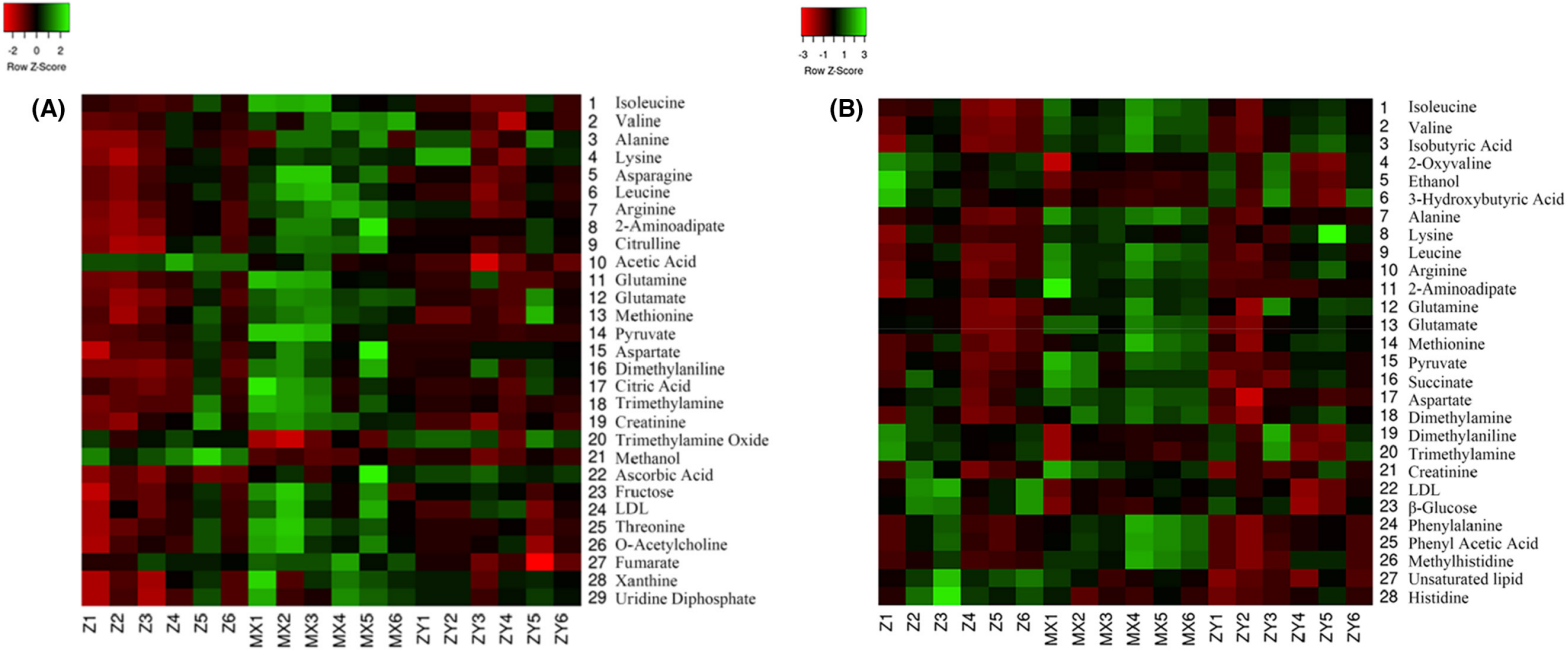
Darker the colour, the higher the metabolite content. The greener the colour, the lower the metabolite content. (A) plasma group; (B) liver group

Heat map is to realize the visualization of data by scaling and colour gradient, which is represented by data matrix. The characteristic is that both can retain the large difference and can show the small difference. Typically, metabolites involved in the same metabolic pathway have similar functions. In the figure, the abscissa represents experimental groups and the ordinate represents metabolites. The shade of the colour represents the level of the metabolite content, the redder the colour represents the higher the metabolite content, and the greener the colour represents the lower the metabolite content. It can be seen from the heat map that metabolites are well distinguished among the groups. The plasma and liver samples are shown in Figure 7.

### 3.5 UPLC‐MS results

The metabolites in plasma and liver detected by UPLC‐MS are shown in Tables S3 and S4, respectively. Total ion chromatogram (TIC) of plasma and liver are shown in Figure S5 (A._1_, A._2_).

#### 3.5.1 Data analysis

##### Global PCA analysis

**FIGURE 8:**
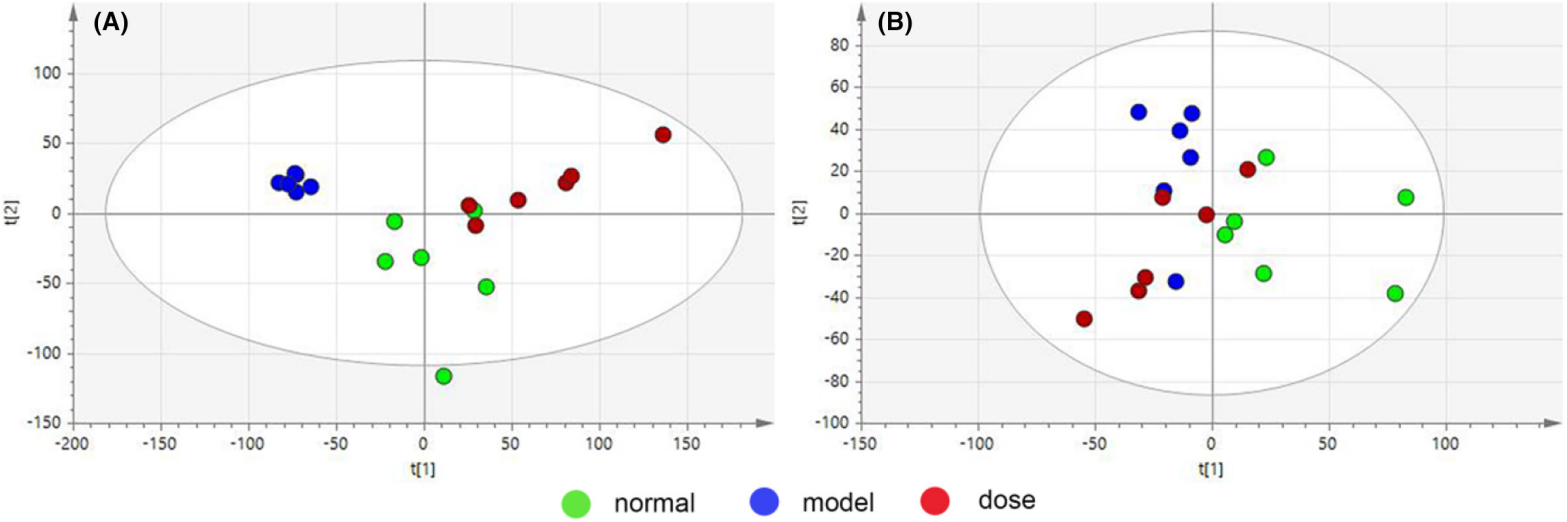
Overall PCA map obtained by UPLC‐MS technique, (A) plasma group *R* ^*2*^ *X* = 0.528, *Q* ^*2*^ = 0.214; (B) liver group *R* ^*2*^ *X* = 0.421, *Q* ^*2*^ = 0.0811

PCA images of plasma and liver are shown in Figure 8, respectively. Plasma sample *R* ^*2*^ *X* = 0.528, *Q* ^*2*^ = 0.214. Liver sample *R* ^*2*^ *X* = 0.421, *Q* ^*2*^ = 0.0811. Significant systemic metabolic differences were observed between these groups.

##### OPLS‐DA score plot

**FIGURE 9:**
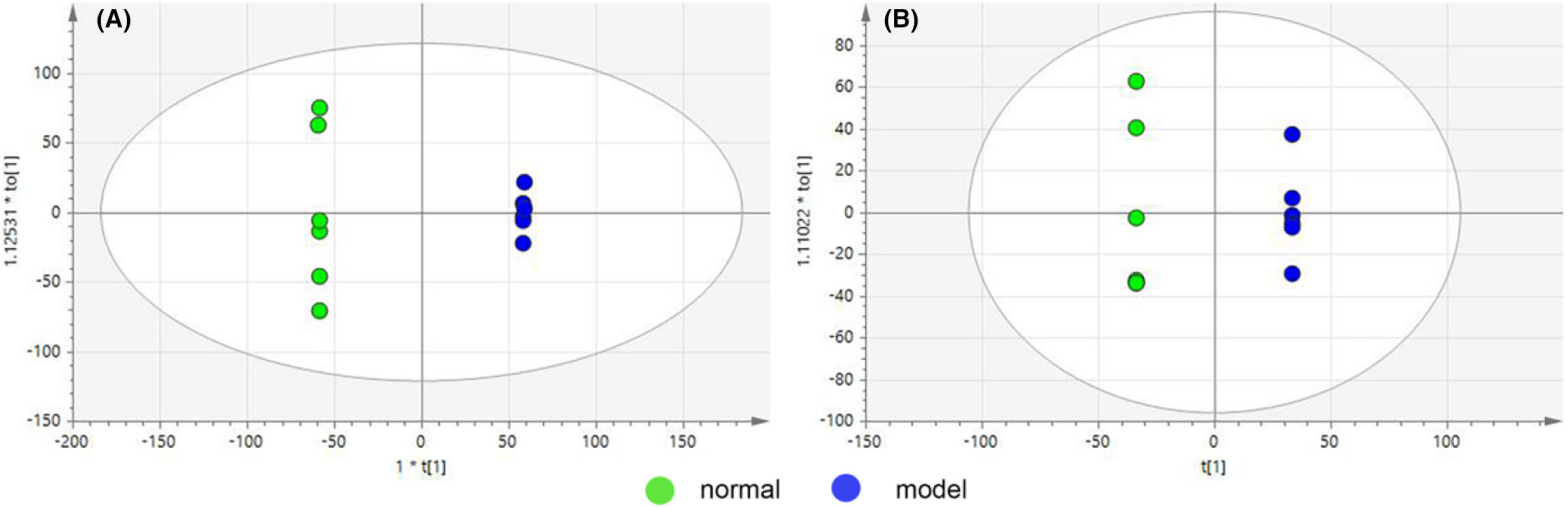
OPLS‐DA score map, obtained by UPLC‐MS, compares the normal group with the model group. (A) plasma group *R* ^*2*^ *X* = 0.685, *R* ^*2*^ *Y* = 1, *Q* ^*2*^ = 0.904; (B) liver group *R* ^*2*^ *X* = 0.628, *R* ^*2*^ *Y* = 1, *Q* ^*2*^ = 0.788

The reasons are as described in 3.4.1.2. The plasma and liver samples were shown in Figure 9. Plasma samples of normal and model group *R* ^*2*^ *X* = 0.685, *R* ^*2*^ *Y* = 1, *Q* ^*2*^ = 0.904. Liver samples in normal and model group *R* ^*2*^ *X* = 0.628, *R* ^*2*^ *Y* = 1, *Q* ^*2*^ = 0.788.

##### OPLS‐DA loading plot

**FIGURE 10:**
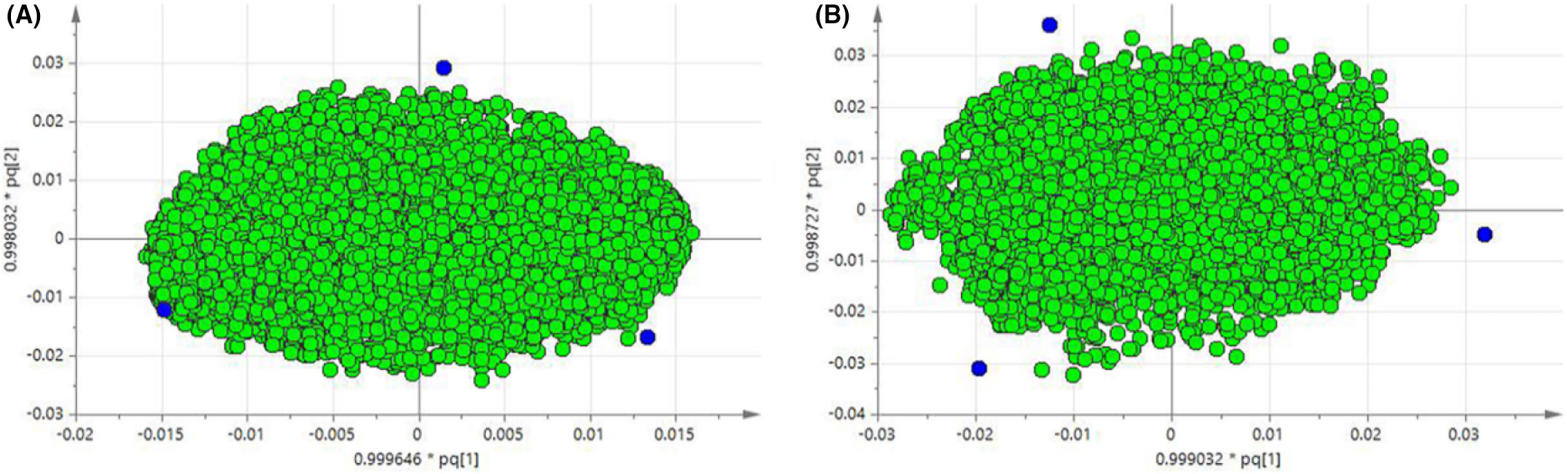
In the Loading diagram obtained by UPLC‐MS, each point represents a metabolite in the sample. Metabolites farther from the origin are considered to contribute more to sample classification. (A) plasma group; (B) liver group

The reasons are as described in 3.4.1.3. The plasma and liver samples were shown in Figure 10.

##### Heat map

**FIGURE 11:**
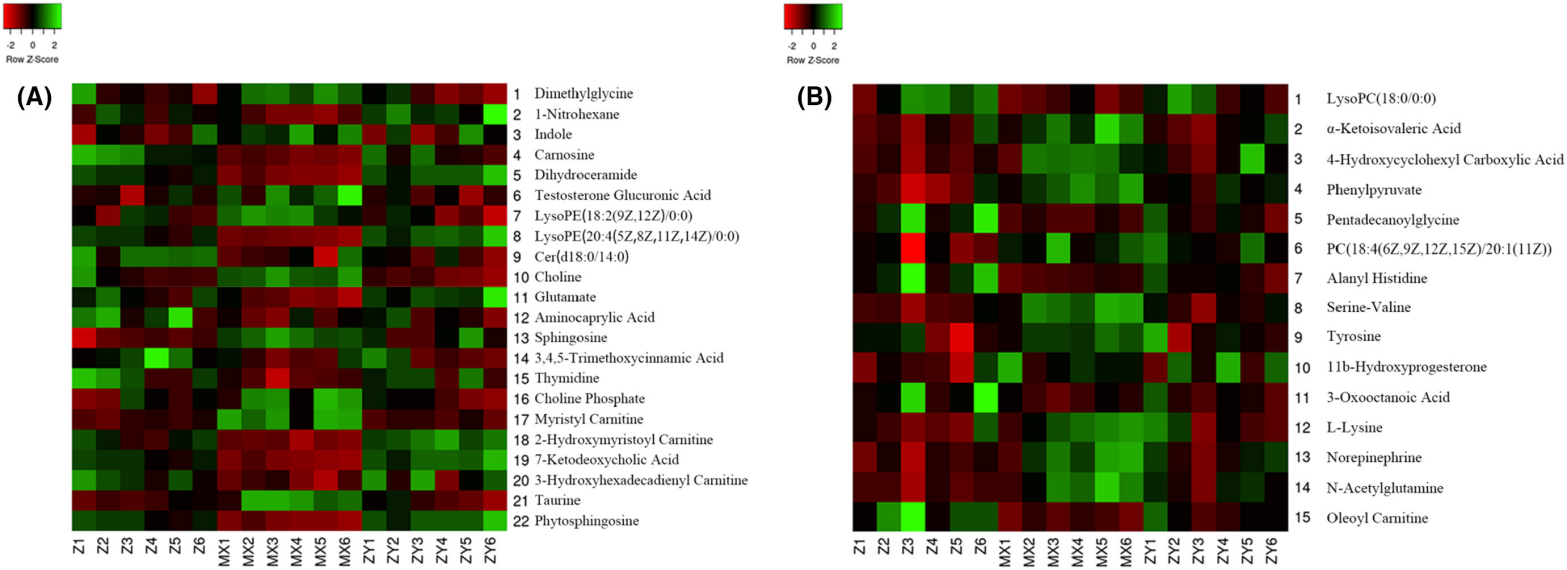
Darker the colour, the higher the metabolite content. The greener the colour, the lower the metabolite content. (A) plasma group; (B) liver group

The reasons are as described in 3.4.1.4. The plasma and liver samples were shown in Figure 11.

#### 3.5.2 Confirmation of the UPLC‐MS analytical method

The methodological validation of all results was less than ±15%, indicating that the established analytical method meets the requirements for the analysis of biological samples, and the results are shown in Table S6.

### 3.6 Analysis of biological significance

The variation trend of metabolites detected in plasma and liver among each group and the metabolic pathways involved are shown in Table S5 (A._1_, A._2_, B._1_, B._2_). Corresponding topologies of plasma and liver are shown in Figures S1 and S2 respectively. The most affected metabolic pathways in the figure are all related to amino acid metabolism. The correlation between various metabolic pathways and metabolites is shown in Figure S3.

Plasma can induce 51 biomarkers such as aspartic acid, alanine, asparagine, citric acid, fumaric acid, glutamine, glutamic acid, threonine, pyruvate, citrulline and sphingosine. Liver induced aspartic acid, alanine, succinic acid, phenylalanine, phenylpyruvate, glutamic acid, glutamine, histidine, serine, arginine, tyrosine and other 43 biomarkers. The combination of plasma and liver biomarkers found that it mainly affects alanine, aspartic acid and glutamate metabolism, phenylalanine metabolism, phenylalanine, tyrosine and tryptophan biosynthesis, and D‐glutathione and D‐glutamate metabolism, histidine metabolism, glycine, serine and threonine metabolism, arginine biosynthesis, tyrosine metabolism, taurine and hypotaurine metabolism, pyruvate metabolism and sphingolipid metabolism. It is found that PEG_2K_‐FIbu/NIC can affect the changes of many metabolites, including amino acid metabolism, glucose metabolism, lipid metabolism, purine metabolism, mainly affecting amino acid metabolism.

Amino acid metabolism: Aspartic acid is reported to play a role in the proliferation of cancer cells. The elevated level of aspartic acid in the blood of liver cancer patients may be due to the nitrogen supply of aspartic acid in the urea cycle.^11^ Asparagine is the only amino acid in the cell that is not catabolized. Removing asparagine from plasma is an effective anticancer measure.^12^ Tumour cells reprogram energy metabolism by promoting glycolysis and inhibiting the TCA cycle. The accumulation of succinic acid and citric acid, intermediate products of TCA cycle, in liver cancer may be related to the obstruction of TCA cycle. TCA cycle not only provides citric acid, but also provides metabolic precursors for the biosynthesis of aspartic acid and asparagine. In order to maintain the activity of amino acids, proliferating cells rely on glutamine supplementation to maintain the integrity of TCA cycle, and the reduction of glutamine will lead to TCA cycle disturbance, resulting in cell death.^12^

Compared with other liver diseases, amino acid metabolism of liver cancer was significantly altered. In particular, high levels of aromatic amino acids such as phenylalanine, which are abundant in acute stage proteins, are elevated by inflammation. It is also believed to be the mechanism of liver disease. It has been reported that the level of phenylalanine in patients with liver cancer is significantly higher than that in healthy people. Phenylalanine is considered as a biomarker of cancer cachexia.^13^, ^14^, ^15^

Glycometabolism: The increased blood pyruvate content may be due to the decreased amount of pyruvate entering TCA circulation or the increased anaerobic respiration of cells.^16^ Glucose is rapidly consumed in tumour cells through a process called glycolysis, and the decrease in glucose and the increase in pyruvate, possibly as a result of reduced mitochondrial respiration, are both signs of the Warburg effect, a specific feature of tumour metabolism. Cancer is involved in higher glycolysis energy expenditure.^17^, ^18^ Pyruvate was metabolized to produce acetic acid and ethanol, and the decrease of acetic acid and ethanol indicated that the mitochondrial TCA cycle in liver was impaired. The decrease of acetic acid content is also related to the increase of fatty acid intake.^19^

Lipid metabolism: Sphingolipids are the main components of cell membrane and play an important role in cell proliferation and apoptosis. Sphingomyelin is synthesized from serine, which can be converted to ceramide (Cer).^20^ Ceramide is the second messenger of apoptosis and senescence in hepatocellular carcinoma. In hepatocellular carcinoma, a decrease in Cer content leads to a decrease in apoptosis, which is associated with high tumour proliferation. Ceramide is broken down by ceramidase into sphingosine, so sphingosine levels are elevated. Ceramide is a central molecule in sphingolipid metabolism, so it plays a role in many diseases.^21^ Sphingosine is a potential marker of liver cancer and can be converted to sphingosine. The decrease of sphingosine in plants indicates a decrease in the synthesis of these metabolites, or they are consumed quickly and sphingosine synthesis is increased.^22^ Sphingosine may induce apoptosis by inhibiting protein kinase C and calmodulin‐dependent enzymes in the nucleus.^23^ Sphingosine has a high predictive effect on liver cancer.^24^

In summary, there were significant differences between the model group and the dosed group and the normal group; we found that these biomarkers will help us prevent liver cancer in the clinic.

## 4 CONCLUSION

It was found that PEG_2K_‐FIbu/NIC had an inhibitory effect on HepG2 cells. Moreover, it can promote apoptosis of HepG2 cells and block S and G2/M phase of cell cycle. The metabolomics of plasma and liver of normal group, model group and administration group were studied by UPLC‐MS and ^1^H‐NMR. Through data integration, related biomarkers were found and related metabolic pathways were found. Thus, the anti‐tumour mechanism of PEG_2K_‐FIbu/NIC micelles was analysed. The results showed that the drug affected a variety of metabolic pathways, such as alanine and glutamic acid and aspartic acid metabolism, phenylalanine metabolism, phenylalanine, tyrosine and tryptophan biosynthesis, D‐glutathione and D‐glutamic acid metabolism, histidine metabolism, glycine, serine and threonine metabolism, arginine biosynthesis, tyrosine metabolism, taurine and low taurine metabolism, pyruvate metabolism and lipid metabolism, mainly affecting protein metabolism, so as to play an anti‐tumour effect.

## AUTHOR CONTRIBUTIONS

**Jiarong Hang:** Writing – original draft (equal). **Yu Chen:** Writing – review and editing (equal). **Lukuan Liu:** Formal analysis (equal); writing – original draft (equal). **Liwen Chen:** Software (equal). **Jiqin Fang:** Resources (equal). **Fei Wang:** Formal analysis (equal); writing – original draft (equal). **Miao Wang:** Supervision (equal).

## CONFLICT OF INTEREST

The authors report no conflicts of interest.

## Supporting information


Appendix S1

## Data Availability

The data that supports the findings of this study are available in the supplementary material of this article
