# Supplementary material for: Antitumor effect and metabonomics of niclosamide micelles
Source: J Cell Mol Med. 2022 Aug 3;26(18):4814–24. doi: 10.1111/jcmm.17509 (PMC9465187; doi:10.1111/jcmm.17509)
Supplement: Supplementary file 1 — Appendix S1 [file JCMM-26-4814-s001.docx]

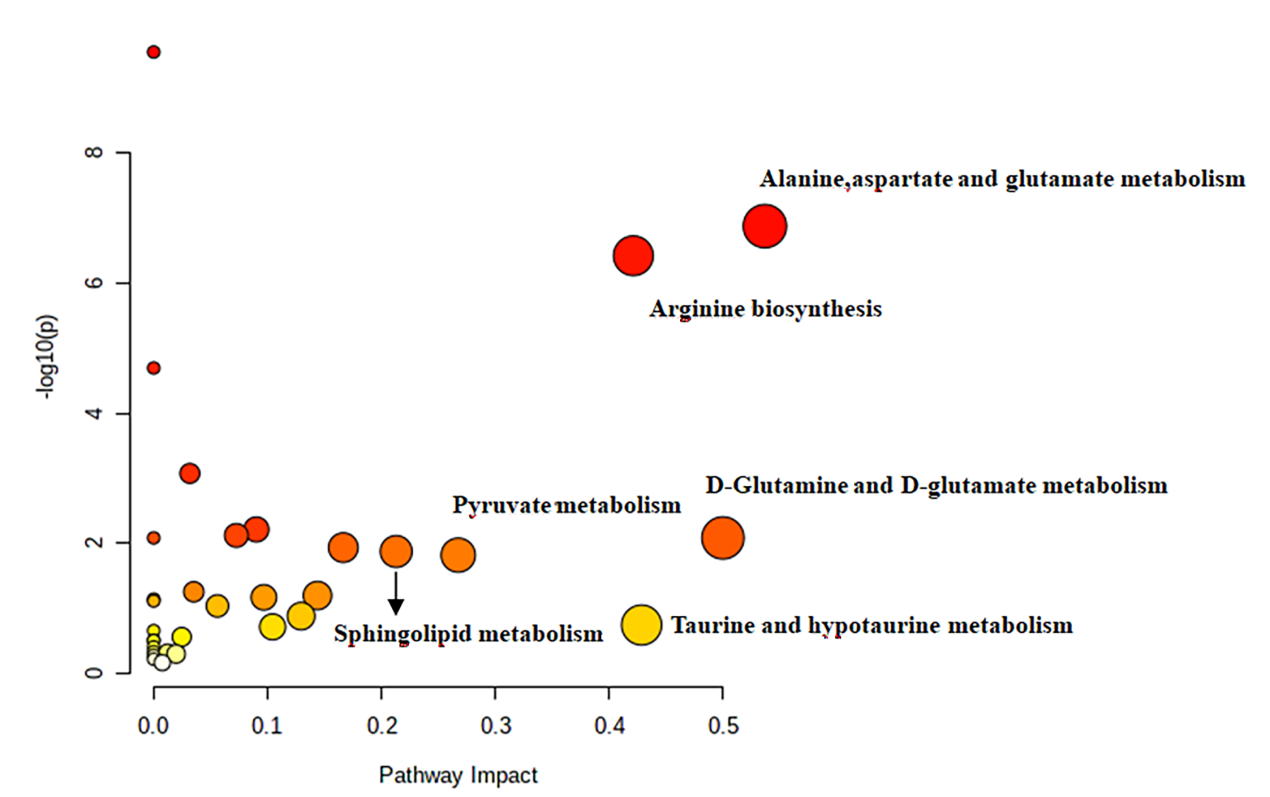


**Figure S1** Topologies of plasma.


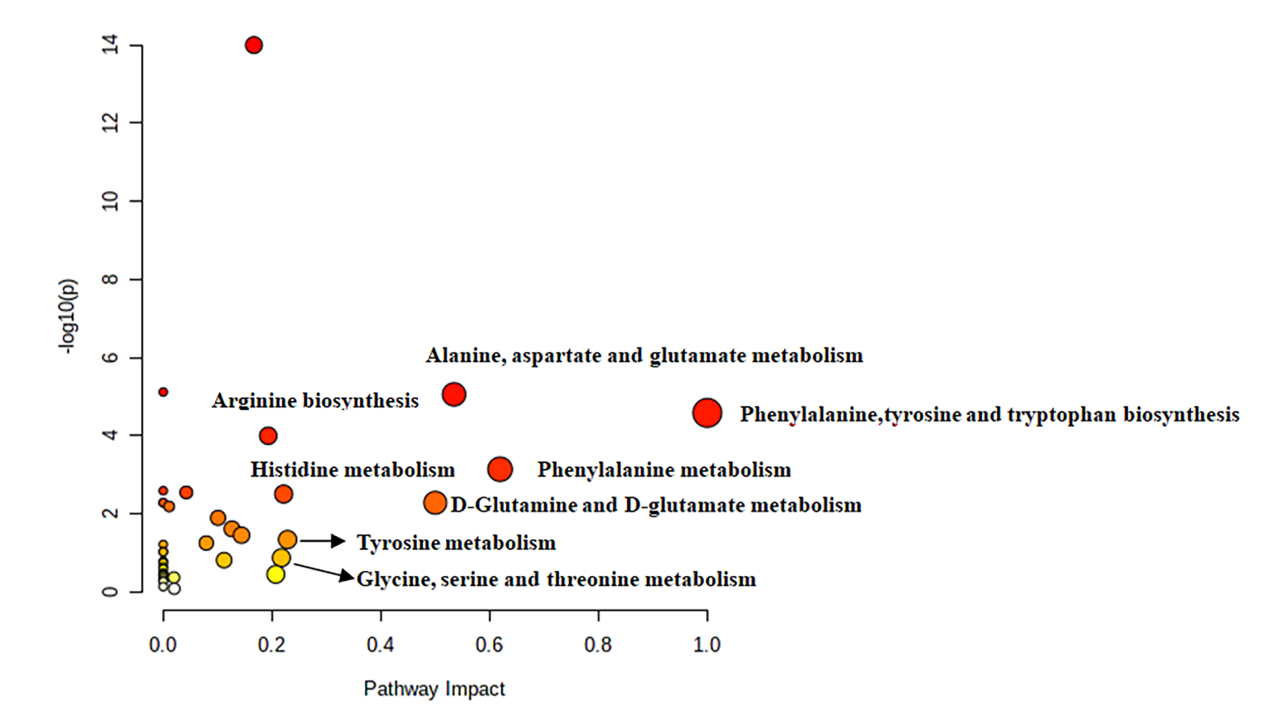


**Figure S2** Topologies of liver.


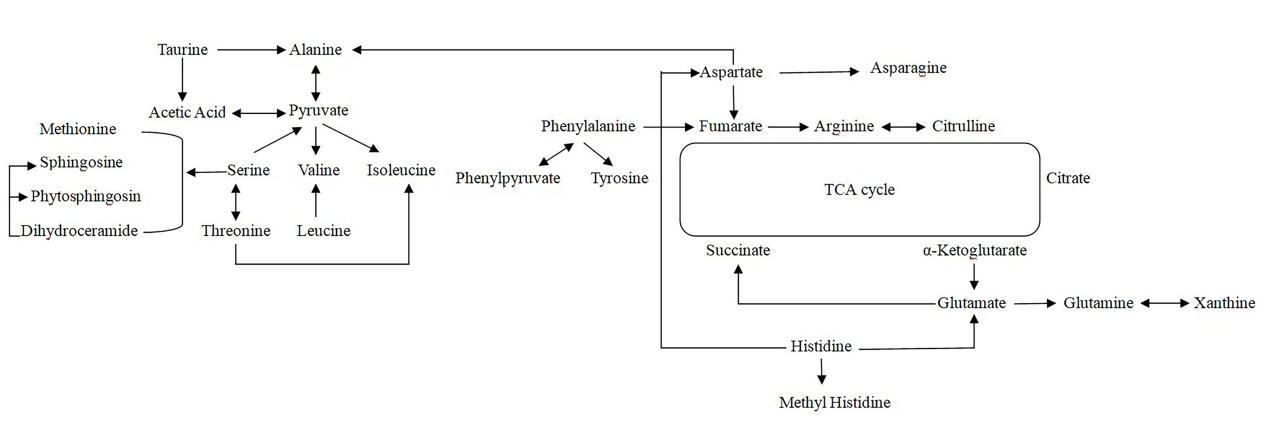


**Figure S3** The correlation between various metabolic pathways and metabolites.


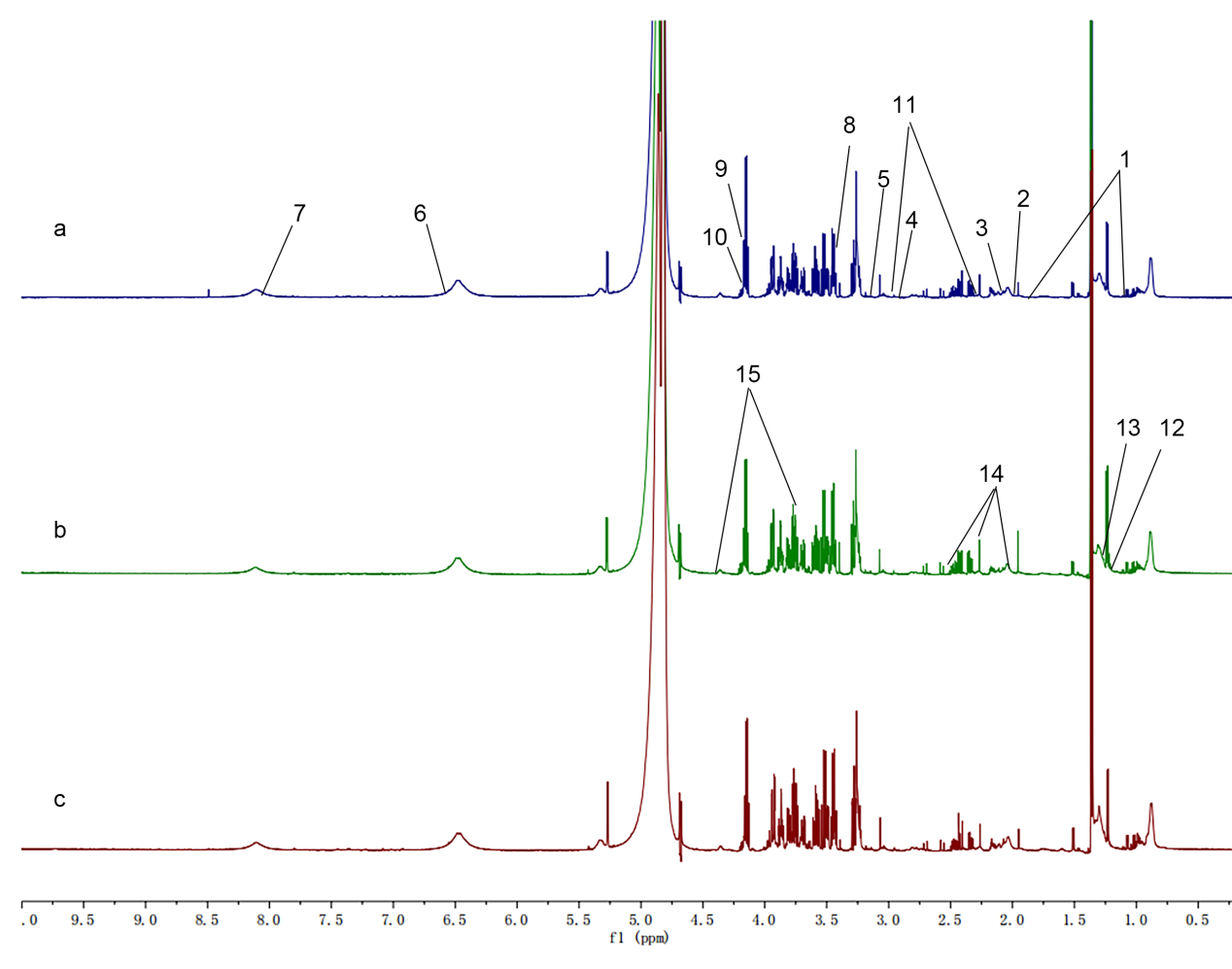


**Figure S4(A._1_)** NMR profiles of plasma samples. (a) PEG_2K_-FIbu/NIC (b) normal (c) model (1) leucine, (2) [citrulline](javascript:;), (3) [acetic](javascript:;) [acid](javascript:;), (4) [dimethylaniline](javascript:;), (5) [creatinine](javascript:;), (6) [fumaric](javascript:;) [acid](javascript:;), (7)  [xanthine](javascript:;), (8) TMAO, (9) [ascorbic](javascript:;) [acid](javascript:;), (10) [fructose](javascript:;), (11) [citric](javascript:;) [acid](javascript:;), (12) [valine](javascript:;), (13) [alanine](javascript:;), (14) [glutamine](javascript:;), (15) [threonine](javascript:;).


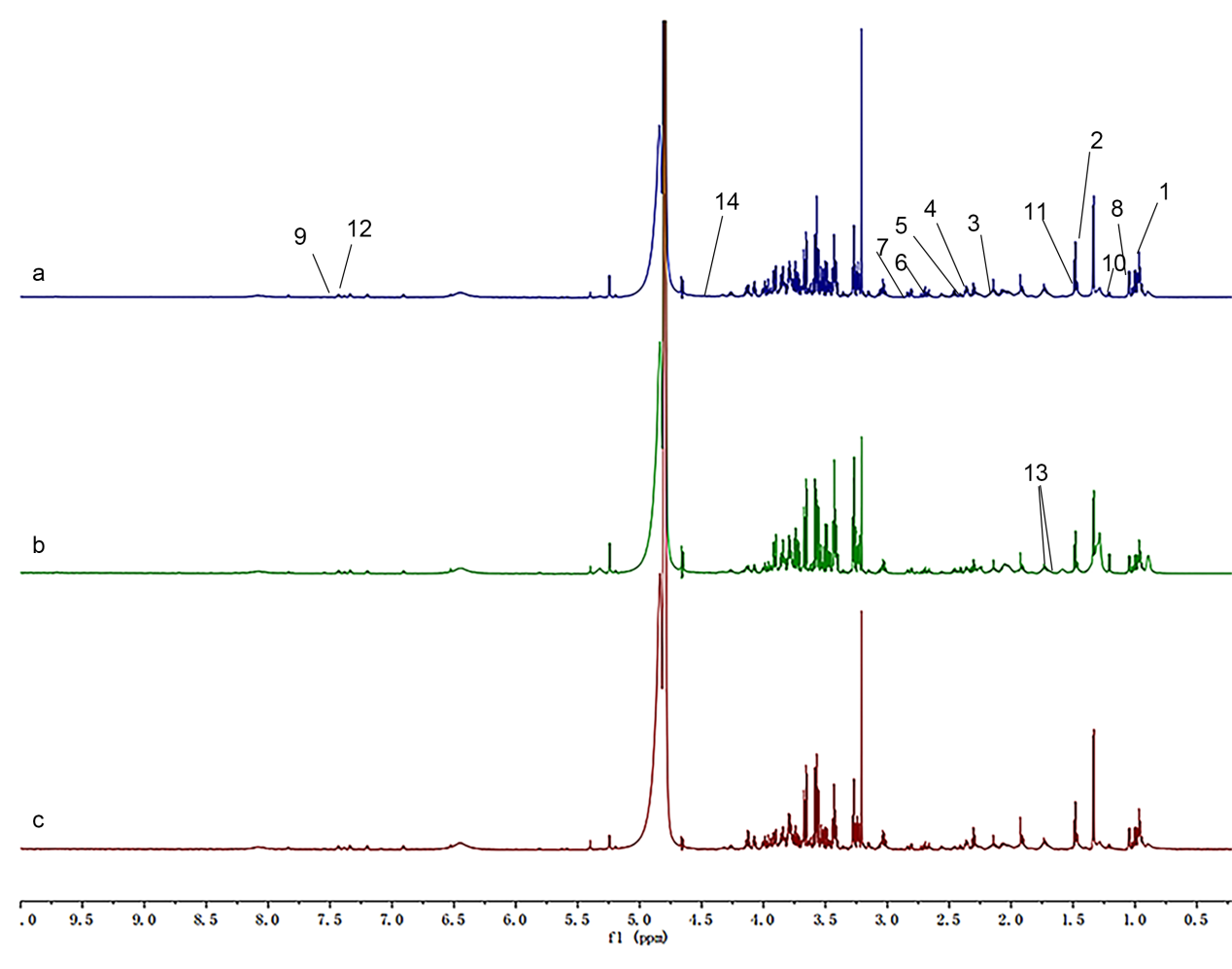


**Figure S4(A._2_)** NMR profiles of liver samples. (a) PEG_2K_-FIbu/NIC (b) model (c) normal (1) [isoleucine](javascript:;), (2) alanine, (3) [methionine](javascript:;), (4) [pyruvic](javascript:;) [acid](javascript:;), (5)  [succinic](javascript:;) [acid](javascript:;), (6) [dimethylamine](javascript:;), (7) [trimethylamine](javascript:;), (8) [isobutyric](javascript:;) [acid](javascript:;), (9) [methylhistidine](javascript:;), (10) 3-hydroxybutyric acid, (11) lysine, (12) phenylalanine, (13) arginine, (14)β- glucose.


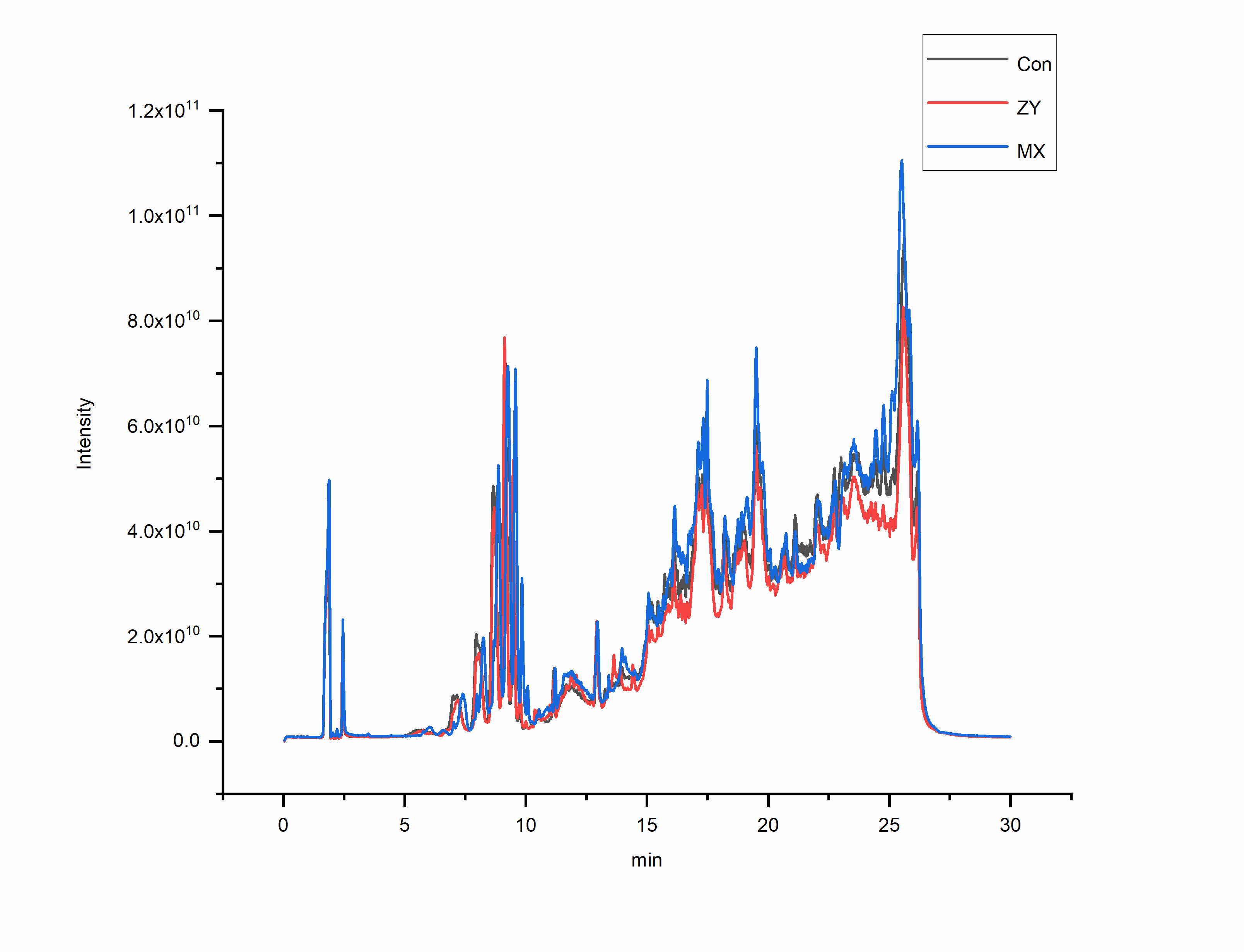


**Figure S5 (A._1_)** TIC of plasma .


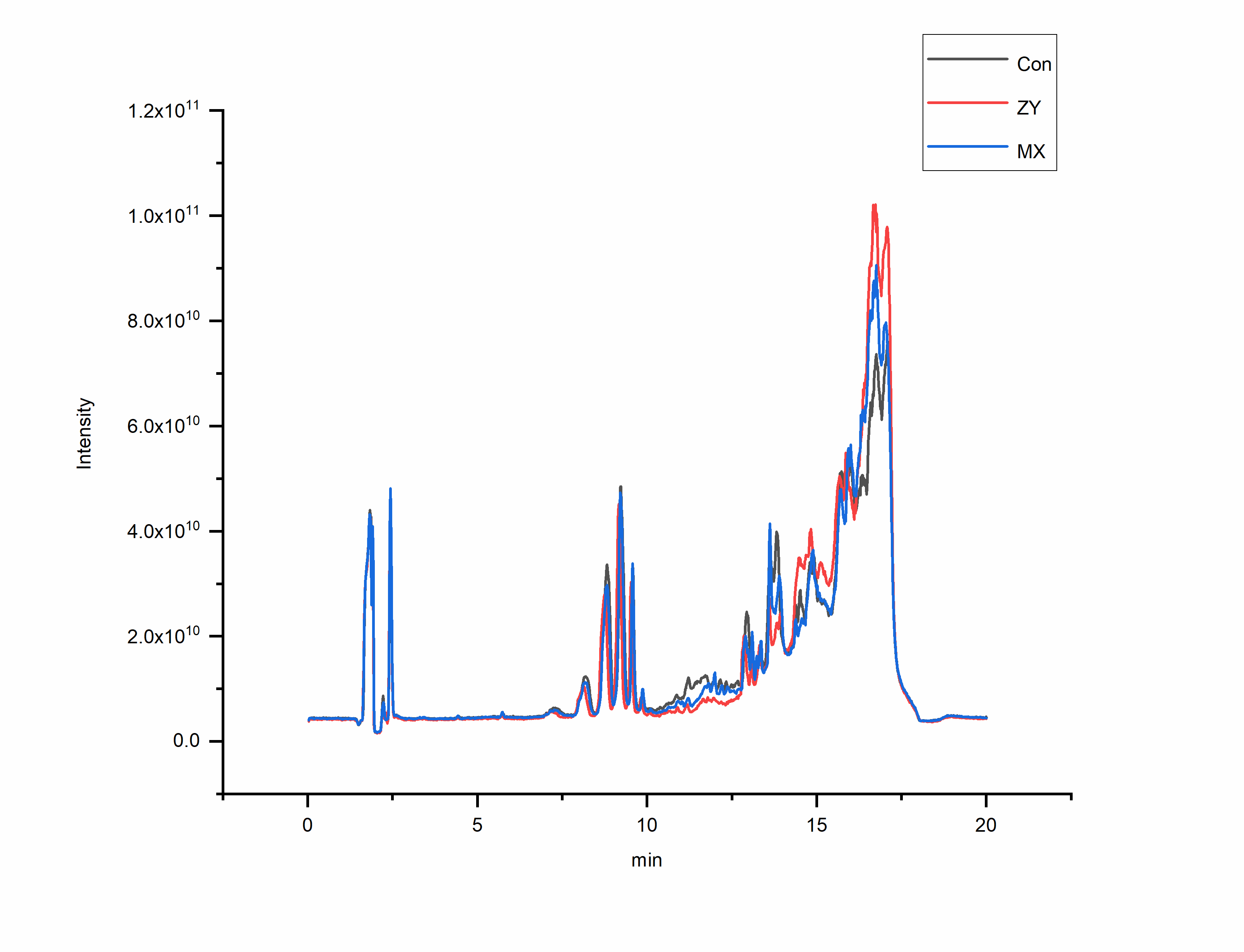


**Figure S5 (A._2_)** TIC of liver.

**Table S1** Metabolites in plasma tested by NMR

| **Number** | [**Metabolite**](javascript:;) | [**Chemical**](javascript:;) [**shift**](javascript:;) **(ppm)** | **Peak belonging** |
| --- | --- | --- | --- |
| 1 | [Isoleucine](javascript:;) | 0.95t | δ-CH_3_ |
| 2 | [Valine](javascript:;) | 1.05 | CH_3_ |
| 3 | [Leucine](javascript:;) | 0.97d 1.72m | δ-CH_3_，β-CH_2_ |
| 4 | [Alanine](javascript:;) | 1.48d | β-CH_3_ |
| 5 | Lysine | 1.51m | β-CH_3_ |
| 6 | Arginine | 1.64m 1.73m | γ-CH_2_ |
| 7 | Asparagine | 1.68m 1.90m 3.76t | γ-CH，α-CH |
| 8 | 2-Aminoadipate | 1.83s | β-CH_2_ |
| 9 | Citrulline | 1.87m | β-CH_2_ |
| 10 | Acetic Acid | 1.94s | CH_3_ |
| 11 | Glutamine | 2.00m 2.14m 2.36m | β-CH_2_,β-CH_2_, γ-CH_2_ |
| 12 | Glutamate | 2.12m 2.46m | α-CH, β-CH_2_ |
| 13 | [Methionine](javascript:;) | 2.16m | CH_3_ |
| 14 | Pyruvate | 2.22s | CH_3_ |
| 15 | Aspartate | 2.60dd 2.80dd | β-CH_2_, α-CH |
| 16 | Dimethylaniline | 2.76s | CH_3_ |
| 17 | Citric Acid | 2.16d 2.81d | CH_2_ |
| 18 | Trimethylamine | 2.88s | CH_3_ |
| 19 | Creatinine | 3.04s | CH_3_ |
| 20 | Methanol | 3.36s | CH_3_ |
| 21 | LDL | 4.24m 0.84t 1.25m | CH_3_(CH_2_)_n_，(CH_2_)_n_ |
| 22 | Threonine | 4.28m 3.58d | α-CH, β-CH |
| 23 | Fumarate | 6.45s | CH = CH |
| 24 | Xanthine | 7.89s | N-CH =N |
| 25 | O-Acetylcholine | 2.14s 3.21s 3.72m 4.55m | α-CH_3_，N-CH_3_，β-CH_2_, α-CH_2_ |
| 26 | Uridine Diphosphate | 4.22m 4.28m 4.37m 5.98d 5.99d 7.96d | CH_2_，4-CH'，3-CH'，2-CH'，5-CH'，6-CH'，1-CH' |
| 27 | Ascorbic Acid | 4.03m | CH |
| 28 | Trimethylamine Oxide | 3.28s | CH_3_ |
| 29 | Fructose | 4.05 | CH |

**Table S2** Metabolites in liver tested by NMR

| **Number** | [**Metabolite**](javascript:;) | [**Chemical**](javascript:;) [**shift**](javascript:;) **(ppm)** | **Peak belonging** |
| --- | --- | --- | --- |
| 1 | [Leucine](javascript:;) | 0.97d 1.72m | δ-CH_3_，β-CH_2_ |
| 2 | [Isoleucine](javascript:;) | 0.95t | δ-CH_3_ |
| 3 | [Valine](javascript:;) | 0.98d 1.03d | CH_3_，CH_3_ |
| 4 | 2-Oxyvaline | 1.12d | CH_3_ |
| 5 | [Alanine](javascript:;) | 1.48d | β-CH_3_ |
| 6 | [Methionine](javascript:;) | 2.16m | CH_3_ |
| 7 | Pyruvate | 2.36s | CH_3_ |
| 8 | Succinate | 2.40s | CH_2_ |
| 9 | Aspartate | 2.68m 2.82m 3.91m | β-CH_2_，β'-CH_2_，α-CH |
| 10 | Dimethylamine | 2.72s | CH_3_ |
| 11 | Dimethylaniline | 2.76s | CH_3_ |
| 12 | Trimethylamine | 2.88s | CH_3_ |
| 13 | Isobutyric Acid | 1.05d | β-CH_3_ |
| 14 | Phenylalanine | 7.32d 7.36d 7.41t | 2-CH, 4-CH, 3-CH |
| 15 | Phenyl Acetic Acid | 7.40d 7.35m | CH_2_ |
| 16 | Methylhistidine | 7.48s | β-CH_2_ |
| 17 | Histidine | 7.10s 7.88s | 5-CH, 3-CH |
| 18 | Unsaturated lipid | 7.05s 7.78s | CHCHCH_2_ |
| 19 | 3-Hydroxybutyric Acid | 1.19d | γ-CH_3_ |
| 20 | Lysine | 1.51m | γ-CH_2_ |
| 21 | Arginine | 1.64m 1.73m | γ-CH_2_ |
| 22 | Glutamine | 2.14m 2.00m 2.36m | β-CH_2_，β-CH_2_，γ-CH_2_ |
| 23 | Glutamate | 2.12m 2.46m | α-CH, β-CH_2_ |
| 24 | LDL | 4.24m 0.84t 1.25m | CH_3_(CH_2_)_n_，（CH_2_)_n_ |
| 25 | 2-Aminoadipate | 1.83s | β-CH_2_ |
| 26 | Ethanol | 1.15d | CH_3_ |
| 27 | Creatinine | 3.04m | CH_3_ |
| 28 | β-Glucose | 4.51 | β-C_1_H |

**Table S3** Metabolites in plasma tested by UPLC-MS

| **Number** | [**Metabolite**](javascript:;) | [**Mass-to-charge**](javascript:;) [**ratio**](javascript:;) |
| --- | --- | --- |
| 1 | Dimethylglycine | 104.0706 |
| 2 | 1-Nitrohexane | 132.1025 |
| 3 | Indole | 134.06 |
| 4 | Carnosine | 227.1139 |
| 5 | Dihydroceramide | 330.3003 |
| 6 | Testosterone Glucuronic Acid | 465.2483 |
| 7 | LysoPE(18:2(9Z,12Z)/0:0) | 478.2928 |
| 8 | LysoPE(20:4(5Z、8Z、11Z、14Z)/0:0) | 502.2928 |
| 9 | Cer(d18:0/14:0) | 512.5037 |
| 10 | Choline | 104.1075 |
| 11 | Glutamate | 148.0604 |
| 12 | Aminocaprylic Acid | 160.1332 |
| 13 | Sphingosine | 300.2897 |
| 14 | 3,4,5-Trimethoxycinnamic Acid | 239.0914 |
| 15 | Thymidine | 243.0975 |
| 16 | Choline Phosphate | 184.0739 |
| 17 | Myristyl Carnitine | 372.3108 |
| 18 | 2-Hydroxymyristoyl Carnitine | 388.3057 |
| 19 | 7-Ketodeoxycholic Acid | 407.2792 |
| 20 | 3-Hydroxyhexadecadienyl Carnitine | 412.3057 |
| 21 | Taurine | 516.2989 |
| 22 | Phytosphingosine | 318.3003 |

**Table S4** Metabolites in liver tested by UPLC-MS

| **Number** | [**Metabolite**](javascript:;) | [**Mass-to-charge**](javascript:;) [**ratio**](javascript:;) |
| --- | --- | --- |
| 1 | LysoPC(18:0/0:0) | 524.3711 |
| 2 | α-Ketoisovaleric Acid | 117.0546 |
| 3 | 4-Hydroxycyclohexyl Carboxylic Acid | 145.0859 |
| 4 | Phenylpyruvate | 165.0546 |
| 5 | Pentadecanoylglycine | 300.2533 |
| 6 | PC(18:4(6Z,9Z,12Z,15Z)/20:1(11Z)) | 808.5851 |
| 7 | Alanyl Histidine | 227.1139 |
| 8 | Serine-Valine | 205.1183 |
| 9 | Tyrosine | 453.3363 |
| 10 | 11b-Hydroxyprogesterone | 331.191 |
| 11 | 3-Oxooctanoic Acid | 159.102 |
| 12 | L-Lysine | 147.1128 |
| 13 | Norepinephrine | 170.0812 |
| 14 | N-Acetylglutamine | 189.087 |
| 15 | Oleoyl Carnitine | 426.3578 |

**Table S5 (A._1_)** Difference markers in plasma

| [**Metabolite**](javascript:;) | **Model to blank ratio** | **Dosing to model ratio** | **VIP** | [**Detection**](javascript:;) [**mode**](javascript:;) |
| --- | --- | --- | --- | --- |
| Isoleucine | ↑* | ↓** | 1.78432 | NMR |
| Valine | ↑** | ↓** | 2.14997 | NMR |
| Leucine | ↑*** | ↓* | 2.5819 | NMR |
| Alanine | ↑* | ↓ | 1.56289 | NMR |
| Lysine | ↑** | ↓ | 2.06667 | NMR |
| Arginine | ↑*** | ↓** | 2.39152 | NMR |
| Asparagine | ↑* | ↓* | 1.73094 | NMR |
| 2-Aminooxalic Acid | ↑** | ↓ | 1.86085 | NMR |
| Citrulline | ↑** | ↓** | 1.81362 | NMR |
| Acetic Acid | ↓** | ↓** | 2.05362 | NMR |
| Glutamine | ↑* | ↓* | 1.84853 | NMR |
| Glutamate | ↑*** | ↓* | 2.3578 | NMR |
| Methionine | ↑* | ↓* | 1.84853 | NMR |
| Pyruvate | ↑* | ↓* | 1.60917 | NMR |
| Aspartate | ↑* | ↓* | 1.6714 | NMR |
| Dimethylaniline | ↑** | ↓ | 1.91265 | NMR |
| Citric Acid | ↑* | ↓* | 1.84853 | NMR |
| Trimethylamine | ↑* | ↓** | 1.57271 | NMR |
| Creatinine | ↑* | ↓*** | 1.47799 | NMR |
| Methanol | ↓*** | ↑ | 2.38745 | NMR |
| LDL | ↑* | ↓ | 1.209 | NMR |
| Threonine | ↑** | ↓** | 1.71878 | NMR |
| Fumarate | ↑* | ↓*** | 1.81645 | NMR |
| Xanthine | ↑* | ↓ | 1.75737 | NMR |
| O-Acetylcholine | ↑* | ↓** | 1.84853 | NMR |
| Uridine Diphosphate | ↑** | ↓** | 1.77165 | NMR |
| Ascorbic Acid | ↑** | ↑ | 1.69653 | NMR |
| Trimethylamine Oxide | ↓* | ↑* | 1.62837 | NMR |
| Fructose | ↑* | ↓ | 1.42235 | NMR |
| Dimethylglycine | ↑* | ↓ | 1.15426 | UPLC-MS |
| 1-Nitrohexane | ↓* | ↑ | 1.17685 | UPLC-MS |
| Indole | ↑* | ↓ | 1.13446 | UPLC-MS |
| Carnosine | ↓*** | ↑* | 1.65789 | UPLC-MS |
| Dihydroceramide | ↓*** | ↑*** | 1.74409 | UPLC-MS |
| Testosterone Glucuronic Acid | ↑* | ↓*** | 1.21901 | UPLC-MS |
| LysoPE（18：2（9Z,12Z）/0:0) | ↑** | ↓** | 1.41587 | UPLC-MS |
| LysoPE（20：4（5Z、8Z、11Z、14Z)/0:0) | ↓*** | ↑*** | 1.72051 | UPLC-MS |
| Cer（d18：0/14：0） | ↓* | ↑ | 1.26901 | UPLC-MS |
| Choline | ↑** | ↓*** | 1.35178 | UPLC-MS |
| Glutamate | ↓* | ↑** | 1.28145 | UPLC-MS |
| Aminocaprylic Acid | ↓* | ↑*** | 1.15603 | UPLC-MS |
| Sphingosine | ↑*** | ↓* | 1.71144 | UPLC-MS |
| 3,4,5-Trimethoxycinnamic Acid | ↓* | ↑ | 1.14335 | UPLC-MS |
| Thymidine | ↓* | ↑* | 1.29374 | UPLC-MS |
| Choline Phosphate | ↑* | ↓* | 1.2265 | UPLC-MS |
| Myristyl Carnitine | ↑*** | ↓*** | 1.67739 | UPLC-MS |
| 2-Hydroxymyristoyl Carnitine | ↓*** | ↑*** | 1.57048 | UPLC-MS |
| 7-Ketodeoxycholic Acid | ↓*** | ↑*** | 1.75348 | UPLC-MS |
| 3-Hydroxyhexadecadienyl Carnitine | ↓*** | ↑* | 1.5225 | UPLC-MS |
| Taurine | ↑*** | ↓** | 1.52926 | UPLC-MS |
| Phytosphingosine | ↓*** | ↑*** | 1.73948 | UPLC-MS |

*** represents *p* <0.001,** represents *p* <0.01,* represents *p* <0.05.

**Table S5 (A._2_)** [Metabolic](javascript:;) [pathways](javascript:;) in plasma

| **Pathway Name** | **Match Status** | **P** | **Impact** |
| --- | --- | --- | --- |
| [Aminoacyl-tRNA biosynthesis](https://www.metaboanalyst.ca/MetaboAnalyst/Secure/pathway/ResultView.xhtml) | [12/48](https://www.metaboanalyst.ca/MetaboAnalyst/Secure/pathway/ResultView.xhtml) | 2.7694E-10 | 0.0 |
| [Alanine, aspartate and glutamate metabolism](https://www.metaboanalyst.ca/MetaboAnalyst/Secure/pathway/ResultView.xhtml) | [8/28](https://www.metaboanalyst.ca/MetaboAnalyst/Secure/pathway/ResultView.xhtml) | 1.3198E-7 | 0.53686 |
| [Arginine biosynthesis](https://www.metaboanalyst.ca/MetaboAnalyst/Secure/pathway/ResultView.xhtml) | [6/14](https://www.metaboanalyst.ca/MetaboAnalyst/Secure/pathway/ResultView.xhtml) | 3.7654E-7 | 0.42132 |
| [Valine, leucine and isoleucine biosynthesis](https://www.metaboanalyst.ca/MetaboAnalyst/Secure/pathway/ResultView.xhtml) | [4/8](https://www.metaboanalyst.ca/MetaboAnalyst/Secure/pathway/ResultView.xhtml) | 2.009E-5 | 0.0 |
| [Glyoxylate and dicarboxylate metabolism](https://www.metaboanalyst.ca/MetaboAnalyst/Secure/pathway/ResultView.xhtml) | [5/32](https://www.metaboanalyst.ca/MetaboAnalyst/Secure/pathway/ResultView.xhtml) | 8.4016E-4 | 0.03175 |
| [Histidine metabolism](https://www.metaboanalyst.ca/MetaboAnalyst/Secure/pathway/ResultView.xhtml) | [3/16](https://www.metaboanalyst.ca/MetaboAnalyst/Secure/pathway/ResultView.xhtml) | 0.0061106 | 0.09016 |
| [Glycine, serine and threonine metabolism](https://www.metaboanalyst.ca/MetaboAnalyst/Secure/pathway/ResultView.xhtml) | [4/33](https://www.metaboanalyst.ca/MetaboAnalyst/Secure/pathway/ResultView.xhtml) | 0.0075488 | 0.0726 |
| [Nitrogen metabolism](https://www.metaboanalyst.ca/MetaboAnalyst/Secure/pathway/ResultView.xhtml) | [2/6](https://www.metaboanalyst.ca/MetaboAnalyst/Secure/pathway/ResultView.xhtml) | 0.008253 | 0.0 |
| [D-Glutamine and D-glutamate metabolism](https://www.metaboanalyst.ca/MetaboAnalyst/Secure/pathway/ResultView.xhtml) | [2/6](https://www.metaboanalyst.ca/MetaboAnalyst/Secure/pathway/ResultView.xhtml) | 0.008253 | 0.5 |
| [Citrate cycle (TCA cycle)](https://www.metaboanalyst.ca/MetaboAnalyst/Secure/pathway/ResultView.xhtml) | [3/20](https://www.metaboanalyst.ca/MetaboAnalyst/Secure/pathway/ResultView.xhtml) | 0.011624 | 0.16653 |
| [Sphingolipid metabolism](https://www.metaboanalyst.ca/MetaboAnalyst/Secure/pathway/ResultView.xhtml) | [3/21](https://www.metaboanalyst.ca/MetaboAnalyst/Secure/pathway/ResultView.xhtml) | 0.013334 | 0.21298 |
| [Pyruvate metabolism](https://www.metaboanalyst.ca/MetaboAnalyst/Secure/pathway/ResultView.xhtml) | [3/22](https://www.metaboanalyst.ca/MetaboAnalyst/Secure/pathway/ResultView.xhtml) | 0.015181 | 0.26749 |
| [Glycerophospholipid metabolism](https://www.metaboanalyst.ca/MetaboAnalyst/Secure/pathway/ResultView.xhtml) | [3/36](https://www.metaboanalyst.ca/MetaboAnalyst/Secure/pathway/ResultView.xhtml) | 0.055637 | 0.03519 |
| [Arginine and proline metabolism](https://www.metaboanalyst.ca/MetaboAnalyst/Secure/pathway/ResultView.xhtml) | [3/38](https://www.metaboanalyst.ca/MetaboAnalyst/Secure/pathway/ResultView.xhtml) | 0.063579 | 0.14386 |
| [Pyrimidine metabolism](https://www.metaboanalyst.ca/MetaboAnalyst/Secure/pathway/ResultView.xhtml) | [3/39](https://www.metaboanalyst.ca/MetaboAnalyst/Secure/pathway/ResultView.xhtml) | 0.06774 | 0.09676 |
| [Valine, leucine and isoleucine degradation](https://www.metaboanalyst.ca/MetaboAnalyst/Secure/pathway/ResultView.xhtml) | [3/40](https://www.metaboanalyst.ca/MetaboAnalyst/Secure/pathway/ResultView.xhtml) | 0.072023 | 0.0 |
| [Pantothenate and CoA biosynthesis](https://www.metaboanalyst.ca/MetaboAnalyst/Secure/pathway/ResultView.xhtml) | [2/19](https://www.metaboanalyst.ca/MetaboAnalyst/Secure/pathway/ResultView.xhtml) | 0.077031 | 0.0 |
| [beta-Alanine metabolism](https://www.metaboanalyst.ca/MetaboAnalyst/Secure/pathway/ResultView.xhtml) | [2/21](https://www.metaboanalyst.ca/MetaboAnalyst/Secure/pathway/ResultView.xhtml) | 0.091769 | 0.05597 |
| [Glycolysis / Gluconeogenesis](https://www.metaboanalyst.ca/MetaboAnalyst/Secure/pathway/ResultView.xhtml) | [2/26](https://www.metaboanalyst.ca/MetaboAnalyst/Secure/pathway/ResultView.xhtml) | 0.13169 | 0.1295 |
| [Taurine and hypotaurine metabolism](https://www.metaboanalyst.ca/MetaboAnalyst/Secure/pathway/ResultView.xhtml) | [1/8](https://www.metaboanalyst.ca/MetaboAnalyst/Secure/pathway/ResultView.xhtml) | 0.18047 | 0.42857 |
| [Cysteine and methionine metabolism](https://www.metaboanalyst.ca/MetaboAnalyst/Secure/pathway/ResultView.xhtml) | [2/33](https://www.metaboanalyst.ca/MetaboAnalyst/Secure/pathway/ResultView.xhtml) | 0.19271 | 0.10446 |
| [Biotin metabolism](https://www.metaboanalyst.ca/MetaboAnalyst/Secure/pathway/ResultView.xhtml) | [1/10](https://www.metaboanalyst.ca/MetaboAnalyst/Secure/pathway/ResultView.xhtml) | 0.22038 | 0.0 |
| [Tyrosine metabolism](https://www.metaboanalyst.ca/MetaboAnalyst/Secure/pathway/ResultView.xhtml) | [2/42](https://www.metaboanalyst.ca/MetaboAnalyst/Secure/pathway/ResultView.xhtml) | 0.27528 | 0.02463 |
| [Butanoate metabolism](https://www.metaboanalyst.ca/MetaboAnalyst/Secure/pathway/ResultView.xhtml) | [1/15](https://www.metaboanalyst.ca/MetaboAnalyst/Secure/pathway/ResultView.xhtml) | 0.31205 | 0.0 |
| [Nicotinate and nicotinamide metabolism](https://www.metaboanalyst.ca/MetaboAnalyst/Secure/pathway/ResultView.xhtml) | [1/15](https://www.metaboanalyst.ca/MetaboAnalyst/Secure/pathway/ResultView.xhtml) | 0.31205 | 0.0 |
| [Selenocompound metabolism](https://www.metaboanalyst.ca/MetaboAnalyst/Secure/pathway/ResultView.xhtml) | [1/2](https://www.metaboanalyst.ca/MetaboAnalyst/Secure/pathway/ResultView.xhtml)0 | 0.39319 | 0.0 |
| [Lysine degradation](https://www.metaboanalyst.ca/MetaboAnalyst/Secure/pathway/ResultView.xhtml) | [1/25](https://www.metaboanalyst.ca/MetaboAnalyst/Secure/pathway/ResultView.xhtml) | 0.46498 | 0.0 |
| [Purine metabolism](https://www.metaboanalyst.ca/MetaboAnalyst/Secure/pathway/ResultView.xhtml) | [2/65](https://www.metaboanalyst.ca/MetaboAnalyst/Secure/pathway/ResultView.xhtml) | 0.47937 | 0.01217 |
| [Glutathione metabolism](https://www.metaboanalyst.ca/MetaboAnalyst/Secure/pathway/ResultView.xhtml) | [1/28](https://www.metaboanalyst.ca/MetaboAnalyst/Secure/pathway/ResultView.xhtml) | 0.50402 | 0.01966 |
| [Porphyrin and chlorophyll metabolism](https://www.metaboanalyst.ca/MetaboAnalyst/Secure/pathway/ResultView.xhtml) | [1/30](https://www.metaboanalyst.ca/MetaboAnalyst/Secure/pathway/ResultView.xhtml) | 0.52848 | 0.0 |
| [Amino sugar and nucleotide sugar metabolism](https://www.metaboanalyst.ca/MetaboAnalyst/Secure/pathway/ResultView.xhtml) | [1/37](https://www.metaboanalyst.ca/MetaboAnalyst/Secure/pathway/ResultView.xhtml) | 0.6052 | 0.0 |
| [Primary bile acid biosynthesis](https://www.metaboanalyst.ca/MetaboAnalyst/Secure/pathway/ResultView.xhtml) | [1/46](https://www.metaboanalyst.ca/MetaboAnalyst/Secure/pathway/ResultView.xhtml) | 0.68617 | 0.00758 |

**Table S5 (B._1_)** Difference markers in liver

| [**Metabolite**](javascript:;) | **Model to blank ratio** | **Dosing to model ratio** | **VIP** | [**Detection**](javascript:;) [**mode**](javascript:;) |
| --- | --- | --- | --- | --- |
| Leucine | ↑*** | ↓*** | 1.84588 | NMR |
| Isoleucine | ↑*** | ↓* | 1.80066 | NMR |
| Valine | ↑*** | ↓* | 1.825161.65686 | NMR |
| 2-Oxyvaline | ↓* | ↑ | 1.46256 | NMR |
| Alanine | ↑*** | ↓*** | 1.97019 | NMR |
| Methionine | ↑** | ↓ | 1.54226 | NMR |
| Pyruvate | ↑** | ↓** | 1.7857 | NMR |
| Succinate | ↑* | ↓* | 1.38682 | NMR |
| Aspartate | ↑** | ↓*** | 1.63717 | NMR |
| Dimethylamine | ↑** | ↓* | 1.65779 | NMR |
| Dimethylaniline | ↓* | ↑ | 1.58974 | NMR |
| Trimethylamine | ↓* | ↑ | 1.57658 | NMR |
| Isobutyric Acid | ↑** | ↓ | 1.68947 | NMR |
| Phenylalanine | ↑* | ↓** | 1.38529 | NMR |
| Phenyl Acetic Acid | ↑* | ↓*** | 1.41632 | NMR |
| Methylhistidine | ↑** | ↓** | 1.73284 | NMR |
| Histidine | ↓* | ↓ | 1.37515 | NMR |
| Unsaturated lipid | ↓* | ↓* | 1.24377 | NMR |
| 3-Hydroxybutyric Acid | ↓* | ↑ | 1.42652 | NMR |
| Lysine | ↑** | ↑ | 1.57497 | NMR |
| Arginine | ↑*** | ↓* | 1.86865 | NMR |
| Glutamine | ↑** | ↓** | 1.7857 | NMR |
| Glutamate | ↑** | ↓** | 1.76301 | NMR |
| LDL | ↓* | ↓ | 1.40501 | NMR |
| 2-Aminooxalic Acid | ↑* | ↓** | 1.44535 | NMR |
| Ethanol | ↓* | ↑ | 1.58053 | NMR |
| Creatinine | ↑* | ↓ | 1.31684 | NMR |
| β-Glucose | ↓* | ↑ | 1.57411 | NMR |
| LysoPC(18:0/0:0) | ↓* | ↑* | 1.66192 | UPLC-MS |
| α-Ketoisovaleric Acid | ↑* | ↓* | 1.61517 | UPLC-MS |
| 4-Hydroxycyclohexyl Carboxylic Acid | ↑** | ↓ | 1.76828 | UPLC-MS |
| Phenylpyruvate | ↑** | ↓* | 1.88699 | UPLC-MS |
| Pentadecanoylglycine | ↓* | ↑ | 1.59253 | UPLC-MS |
| PC(18:4(6Z,9Z,12Z,15Z)/20:1(11Z)) | ↑* | ↓ | 1.52048 | UPLC-MS |
| Alanyl Histidine | ↓* | ↑ | 1.64469 | UPLC-MS |
| Serine-Valine | ↑*** | ↓** | 2.00169 | UPLC-MS |
| Tyrosine | ↑* | ↓ | 1.38386 | UPLC-MS |
| 11b-Hydroxyprogesterone | ↑* | ↓ | 1.37151 | UPLC-MS |
| 3-Oxooctanoic Acid | ↓* | ↑* | 1.28926 | UPLC-MS |
| L-Lysine | ↑* | ↓ | 1.56187 | UPLC-MS |
| Norepinephrine | ↑** | ↓ | 1.74622 | UPLC-MS |
| N-Acetylglutamine | ↑** | ↓ | 1.82753 | UPLC-MS |
| Oleoyl Carnitine | ↓** | ↑* | 1.84177 | UPLC-MS |

*** represents *p* <0.001,** represents *p* <0.01,* represents *p* <0.05.

**Table S5 (B._2_)** [Metabolic](javascript:;) [pathways](javascript:;) in liver

| **Pathway Name** | **Match Status** | **P** | **Impact** |
| --- | --- | --- | --- |
| [Aminoacyl-tRNA biosynthesis](https://www.metaboanalyst.ca/MetaboAnalyst/Secure/pathway/ResultView.xhtml) | [14/48](https://www.metaboanalyst.ca/MetaboAnalyst/Secure/pathway/ResultView.xhtml) | 1.0039E-14 | 0.16667 |
| [Valine, leucine and isoleucine biosynthesis](https://www.metaboanalyst.ca/MetaboAnalyst/Secure/pathway/ResultView.xhtml) | [4/8](https://www.metaboanalyst.ca/MetaboAnalyst/Secure/pathway/ResultView.xhtml) | 7.5852E-6 | 0.0 |
| [Alanine, aspartate and glutamate metabolism](https://www.metaboanalyst.ca/MetaboAnalyst/Secure/pathway/ResultView.xhtml) | [6/28](https://www.metaboanalyst.ca/MetaboAnalyst/Secure/pathway/ResultView.xhtml) | 8.7233E-6 | 0.53446 |
| [Phenylalanine, tyrosine and tryptophan biosynthesis](https://www.metaboanalyst.ca/MetaboAnalyst/Secure/pathway/ResultView.xhtml) | [3/4](https://www.metaboanalyst.ca/MetaboAnalyst/Secure/pathway/ResultView.xhtml) | 2.5874E-5 | 1.0 |
| [Arginine biosynthesis](https://www.metaboanalyst.ca/MetaboAnalyst/Secure/pathway/ResultView.xhtml) | [4/14](https://www.metaboanalyst.ca/MetaboAnalyst/Secure/pathway/ResultView.xhtml) | 1.0E-4 | 0.19289 |
| [Phenylalanine metabolism](https://www.metaboanalyst.ca/MetaboAnalyst/Secure/pathway/ResultView.xhtml) | [3/10](https://www.metaboanalyst.ca/MetaboAnalyst/Secure/pathway/ResultView.xhtml) | 7.1729E-4 | 0.61904 |
| [Butanoate metabolism](https://www.metaboanalyst.ca/MetaboAnalyst/Secure/pathway/ResultView.xhtml) | [3/15](https://www.metaboanalyst.ca/MetaboAnalyst/Secure/pathway/ResultView.xhtml) | 0.0025469 | 0.0 |
| [Glyoxylate and dicarboxylate metabolism](https://www.metaboanalyst.ca/MetaboAnalyst/Secure/pathway/ResultView.xhtml) | [4/32](https://www.metaboanalyst.ca/MetaboAnalyst/Secure/pathway/ResultView.xhtml) | 0.0028159 | 0.04233 |
| [Histidine metabolism](https://www.metaboanalyst.ca/MetaboAnalyst/Secure/pathway/ResultView.xhtml) | [3/16](https://www.metaboanalyst.ca/MetaboAnalyst/Secure/pathway/ResultView.xhtml) | 0.0030938 | 0.22131 |
| [Pantothenate and CoA biosynthesis](https://www.metaboanalyst.ca/MetaboAnalyst/Secure/pathway/ResultView.xhtml) | [3/19](https://www.metaboanalyst.ca/MetaboAnalyst/Secure/pathway/ResultView.xhtml) | 0.005147 | 0.0 |
| [Nitrogen metabolism](https://www.metaboanalyst.ca/MetaboAnalyst/Secure/pathway/ResultView.xhtml) | [2/6](https://www.metaboanalyst.ca/MetaboAnalyst/Secure/pathway/ResultView.xhtml) | 0.0051783 | 0.0 |
| [D-Glutamine and D-glutamate metabolism](https://www.metaboanalyst.ca/MetaboAnalyst/Secure/pathway/ResultView.xhtml) | [2/6](https://www.metaboanalyst.ca/MetaboAnalyst/Secure/pathway/ResultView.xhtml) | 0.0051783 | 0.5 |
| [Valine, leucine and isoleucine degradation](https://www.metaboanalyst.ca/MetaboAnalyst/Secure/pathway/ResultView.xhtml) | [4/40](https://www.metaboanalyst.ca/MetaboAnalyst/Secure/pathway/ResultView.xhtml) | 0.0064232 | 0.01084 |
| [Glycolysis / Gluconeogenesis](https://www.metaboanalyst.ca/MetaboAnalyst/Secure/pathway/ResultView.xhtml) | [3/26](https://www.metaboanalyst.ca/MetaboAnalyst/Secure/pathway/ResultView.xhtml) | 0.012603 | 0.10065 |
| [Cysteine and methionine metabolism](https://www.metaboanalyst.ca/MetaboAnalyst/Secure/pathway/ResultView.xhtml) | [3/33](https://www.metaboanalyst.ca/MetaboAnalyst/Secure/pathway/ResultView.xhtml) | 0.024141 | 0.1263 |
| [Arginine and proline metabolism](https://www.metaboanalyst.ca/MetaboAnalyst/Secure/pathway/ResultView.xhtml) | [3/38](https://www.metaboanalyst.ca/MetaboAnalyst/Secure/pathway/ResultView.xhtml) | 0.034979 | 0.14386 |
| [Tyrosine metabolism](https://www.metaboanalyst.ca/MetaboAnalyst/Secure/pathway/ResultView.xhtml) | [3/42](https://www.metaboanalyst.ca/MetaboAnalyst/Secure/pathway/ResultView.xhtml) | 0.045196 | 0.22844 |
| [Citrate cycle (TCA cycle)](https://www.metaboanalyst.ca/MetaboAnalyst/Secure/pathway/ResultView.xhtml) | [2/20](https://www.metaboanalyst.ca/MetaboAnalyst/Secure/pathway/ResultView.xhtml) | 0.055453 | 0.07907 |
| [beta-Alanine metabolism](https://www.metaboanalyst.ca/MetaboAnalyst/Secure/pathway/ResultView.xhtml) | [2/21](https://www.metaboanalyst.ca/MetaboAnalyst/Secure/pathway/ResultView.xhtml) | 0.060566 | 0.0 |
| [Synthesis and degradation of ketone bodies](https://www.metaboanalyst.ca/MetaboAnalyst/Secure/pathway/ResultView.xhtml) | [1/5](https://www.metaboanalyst.ca/MetaboAnalyst/Secure/pathway/ResultView.xhtml) | 0.093216 | 0.0 |
| [Linoleic acid metabolism](https://www.metaboanalyst.ca/MetaboAnalyst/Secure/pathway/ResultView.xhtml) | [1/5](https://www.metaboanalyst.ca/MetaboAnalyst/Secure/pathway/ResultView.xhtml) | 0.093216 | 0.0 |
| [Glycine, serine and threonine metabolism](https://www.metaboanalyst.ca/MetaboAnalyst/Secure/pathway/ResultView.xhtml) | [2/33](https://www.metaboanalyst.ca/MetaboAnalyst/Secure/pathway/ResultView.xhtml) | 0.13218 | 0.21707 |
| [Glycerophospholipid metabolism](https://www.metaboanalyst.ca/MetaboAnalyst/Secure/pathway/ResultView.xhtml) | [2/36](https://www.metaboanalyst.ca/MetaboAnalyst/Secure/pathway/ResultView.xhtml) | 0.15228 | 0.11182 |
| [Ubiquinone and other terpenoid-quinone biosynthesis](https://www.metaboanalyst.ca/MetaboAnalyst/Secure/pathway/ResultView.xhtml) | [1/9](https://www.metaboanalyst.ca/MetaboAnalyst/Secure/pathway/ResultView.xhtml) | 0.16169 | 0.0 |
| [Biotin metabolism](https://www.metaboanalyst.ca/MetaboAnalyst/Secure/pathway/ResultView.xhtml) | [1/10](https://www.metaboanalyst.ca/MetaboAnalyst/Secure/pathway/ResultView.xhtml) | 0.17801 | 0.0 |
| [alpha-Linolenic acid metabolism](https://www.metaboanalyst.ca/MetaboAnalyst/Secure/pathway/ResultView.xhtml) | [1/13](https://www.metaboanalyst.ca/MetaboAnalyst/Secure/pathway/ResultView.xhtml) | 0.22514 | 0.0 |
| [Nicotinate and nicotinamide metabolism](https://www.metaboanalyst.ca/MetaboAnalyst/Secure/pathway/ResultView.xhtml) | [1/15](https://www.metaboanalyst.ca/MetaboAnalyst/Secure/pathway/ResultView.xhtml) | 0.25511 | 0.0 |
| [Selenocompound metabolism](https://www.metaboanalyst.ca/MetaboAnalyst/Secure/pathway/ResultView.xhtml) | [1/20](https://www.metaboanalyst.ca/MetaboAnalyst/Secure/pathway/ResultView.xhtml) | 0.3252 | 0.0 |
| [Sphingolipid metabolism](https://www.metaboanalyst.ca/MetaboAnalyst/Secure/pathway/ResultView.xhtml) | [1/21](https://www.metaboanalyst.ca/MetaboAnalyst/Secure/pathway/ResultView.xhtml) | 0.33843 | 0.0 |
| [Pyruvate metabolism](https://www.metaboanalyst.ca/MetaboAnalyst/Secure/pathway/ResultView.xhtml) | [1/22](https://www.metaboanalyst.ca/MetaboAnalyst/Secure/pathway/ResultView.xhtml) | 0.35141 | 0.20684 |
| [Propanoate metabolism](https://www.metaboanalyst.ca/MetaboAnalyst/Secure/pathway/ResultView.xhtml) | [1/23](https://www.metaboanalyst.ca/MetaboAnalyst/Secure/pathway/ResultView.xhtml) | 0.36414 | 0.0 |
| [Lysine degradation](https://www.metaboanalyst.ca/MetaboAnalyst/Secure/pathway/ResultView.xhtml) | [1/25](https://www.metaboanalyst.ca/MetaboAnalyst/Secure/pathway/ResultView.xhtml) | 0.38889 | 0.0 |
| [Glutathione metabolism](https://www.metaboanalyst.ca/MetaboAnalyst/Secure/pathway/ResultView.xhtml) | [1/28](https://www.metaboanalyst.ca/MetaboAnalyst/Secure/pathway/ResultView.xhtml) | 0.42427 | 0.01966 |
| [Porphyrin and chlorophyll metabolism](https://www.metaboanalyst.ca/MetaboAnalyst/Secure/pathway/ResultView.xhtml) | [1/30](https://www.metaboanalyst.ca/MetaboAnalyst/Secure/pathway/ResultView.xhtml) | 0.44675 | 0.0 |
| [Arachidonic acid metabolism](https://www.metaboanalyst.ca/MetaboAnalyst/Secure/pathway/ResultView.xhtml) | [1/36](https://www.metaboanalyst.ca/MetaboAnalyst/Secure/pathway/ResultView.xhtml) | 0.50922 | 0.0 |
| [Pyrimidine metabolism](https://www.metaboanalyst.ca/MetaboAnalyst/Secure/pathway/ResultView.xhtml) | [1/39](https://www.metaboanalyst.ca/MetaboAnalyst/Secure/pathway/ResultView.xhtml) | 0.53784 | 0.0 |
| [Purine metabolism](https://www.metaboanalyst.ca/MetaboAnalyst/Secure/pathway/ResultView.xhtml) | [1/65](https://www.metaboanalyst.ca/MetaboAnalyst/Secure/pathway/ResultView.xhtml) | 0.72683 | 0.0 |
| [Steroid hormone biosynthesis](https://www.metaboanalyst.ca/MetaboAnalyst/Secure/pathway/ResultView.xhtml) | [1/85](https://www.metaboanalyst.ca/MetaboAnalyst/Secure/pathway/ResultView.xhtml) | 0.81886 | 0.02021 |

**Table S6** Stability and repeatability results of the UPLC-MS platform

| Biological  matrices | *t*_R__m/z  (min_Da) | System stability(RSD %) | | Repeatability (RSD %) | | Post-preparative stability(RE %) | | Freeze–thaw  stability(RE %) | |
| --- | --- | --- | --- | --- | --- | --- | --- | --- | --- |
|  |  | *t*_R_ | Area | *t*_R_ | Area | *t*_R_ | Area | *t*_R_ | Area |
| Plasma | 5.6-310.4 | 1.8 | 6.8 | 1.9 | 4.1 | 1.2 | 5.5 | 2.1 | 4.9 |
|  | 9.1-396.3 | 0.9 | 5.2 | 1.2 | 5.0 | 1.6 | 7.0 | 1.3 | 5.0 |
|  | 13.1-452.6 | 2.0 | 7.0 | 1.6 | 5.2 | 1.0 | 9.7 | 1.5 | 7.7 |
|  | 14.5-332.7 | 2.0 | 13.3 | 1.7 | 6.6 | 1.5 | 7.6 | 1.4 | 5.1 |
|  | 16.2-410.8 | 1.9 | 5.8 | 1.9 | 7.9 | 1.0 | 6.6 | 1.8 | 9.6 |
|  | 18.3-513.9 | 1.2 | 8.0 | 1.3 | 12.0 | 1.6 | 6.8 | 1.4 | 6.6 |
| Liver | 2.4-116.5 | 1.1 | 9.4 | 0.6 | 3.1 | 1.3 | 7.1 | 0.9 | 5.5 |
|  | 7.2-450.2 | 0.7 | 2.6 | 1.2 | 8.3 | 1.1 | 9.1 | 1.1 | 3.6 |
|  | 8.8-689.9 | 1.3 | 5.8 | 1.0 | 4.2 | 1.3 | 2.9 | 1.3 | 4.1 |
|  | 9.6-388.6 | 0.9 | 3.2 | 1.3 | 2.5 | 0.9 | 5.2 | 1.2 | 7.1 |
|  | 12.8-279.1 | 1.3 | 4.3 | 0.9 | 5.3 | 1.2 | 4.4 | 1.0 | 2.1 |
|  | 16.7-583.9 | 1.1 | 4.2 | 1.0 | 2.9 | 1.0 | 3.4 | 0.9 | 8.4 |
